# Topology‐Enriched Toughness Enhancement in Quasi‐Periodic Metastructures Featuring Tailorable Strong‐Weak Network

**DOI:** 10.1002/advs.75413

**Published:** 2026-04-20

**Authors:** Tianyu Gao, Genda Wang, Jiabao Bai, Kai Liu

**Affiliations:** ^1^ Department of Engineering Mechanics Zhejiang University Hangzhou Zhejiang China; ^2^ Institute of Advanced Equipment College of Energy Engineering Zhejiang University Hangzhou Zhejiang China; ^3^ School of Traffic & Transportation Engineering Central South University Changsha Hunan China

**Keywords:** architected weak bonds, customized ligaments, fracture toughening, golden‐ratio‐constrained

## Abstract

Thin‐walled cellular structures deliver ultralow density and high specific strength for damage‐tolerant components. Yet periodic lattices may hard‐wire repeating weak features that promote tensile‐catastrophic fracture, whereas aperiodic networks erase this threat by relinquishing geometric modulation. In this work, a quasi‐periodic Dart‐Kite metastructure (QDK) is built, inspired by the order‐disorder synergy of skeletal muscle. Its golden‐ratio‐constrained geometry forms an intrinsic network of strong and weak bonds, enabling simultaneous improvements in strength, toughness and damage tolerance. QDK substantially surpasses the periodic structure, increasing initial fracture resistance and fracture energy by 211% and 133%, and achieving 1.7× higher cyclic energy absorption. These gains arise from a dispersed stress field generated by the diverse unit orientations and connectivity, which enlarge the plastic zone and promote crack deflection, branching, and arrest. Mechanical performance and crack trajectories remain robust across orientations (<2.5% variation). Introducing architected weak bonds, serving a role similar to the muscle Z‐line, provides controllable fracture pathways while preserving structural performance; notably, toughness rises further, with QDK‐D2 achieving a 24% increase. These features establish quasi‐periodicity as a design principle that unlocks the simultaneous realization of mechanical programmability and high fracture resistance, enabling tunable high‐toughness performance across both general and failure‐specific scenarios.

## Graphical abstract

A quasi‐periodic Dart‐Kite (QDK) metastructure with a golden‐ratio‐constrained strong–weak bond network simultaneously enhances strength, toughness, and damage tolerance. Its distributed topology enables predictable, tailorable crack paths for precise fracture control and stable mechanics, demonstrating a high‐performance, controllable architecture with potential for advanced protective applications.

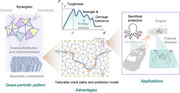

## 1 Introduction

Multi‐cellular structures constitute a versatile class of lightweight structural systems that combine low density with excellent mechanical performance and are readily compatible with large‐scale manufacturing [1, 2]. These features have driven widespread adoption in protective systems and artificial infrastructures [3, 4]. To date, normal design space has been largely charted by periodic motifs—square [5], triangular [6], chiral [7] and honeycomb patterns [8]. Mechanical studies show that under buckling‐governed loading conditions, including compression and bending, the periodic pattern of identical units gives rise to well‐defined deformation modes [9, 10]. The ordered pathways enable effective moduli and in‐plane stiffness and strength that rival those of topologically complex structures [11, 12, 13, 14], including Voronoi [15, 16] and Einstein networks [17], triply periodic minimal surfaces [18, 19, 20] and hierarchical lattices [21, 22]. However, the translational symmetry that leads to predictable collapse sequences also imposes intrinsic limitations. When constructed from comparatively brittle materials or subjected to tension‐dominated loading, periodic structures tend to coherently amplify microstructural damage [23, 24, 25]. Cracks preferentially nucleate at boundary units and, because identical units fail at similar thresholds, propagate rapidly through the network, resulting in abrupt, system‐level brittle failure. This behavior sharply contrasts with the progressive failure modes observed in aperiodic structures. However, stochastic aperiodic structures often exhibit lower peak forces, uncertain configurations and mechanical responses that are difficult to precisely control [26]. This juxtaposition exposes a central dilemma: periodic structures offer controllable configurations and reliable strength but lack toughness, whereas stochastic aperiodic systems provide superior toughness at cost of reduced load‐bearing capacity and increased configurational and mechanical uncertainty.

**FIGURE 1:**
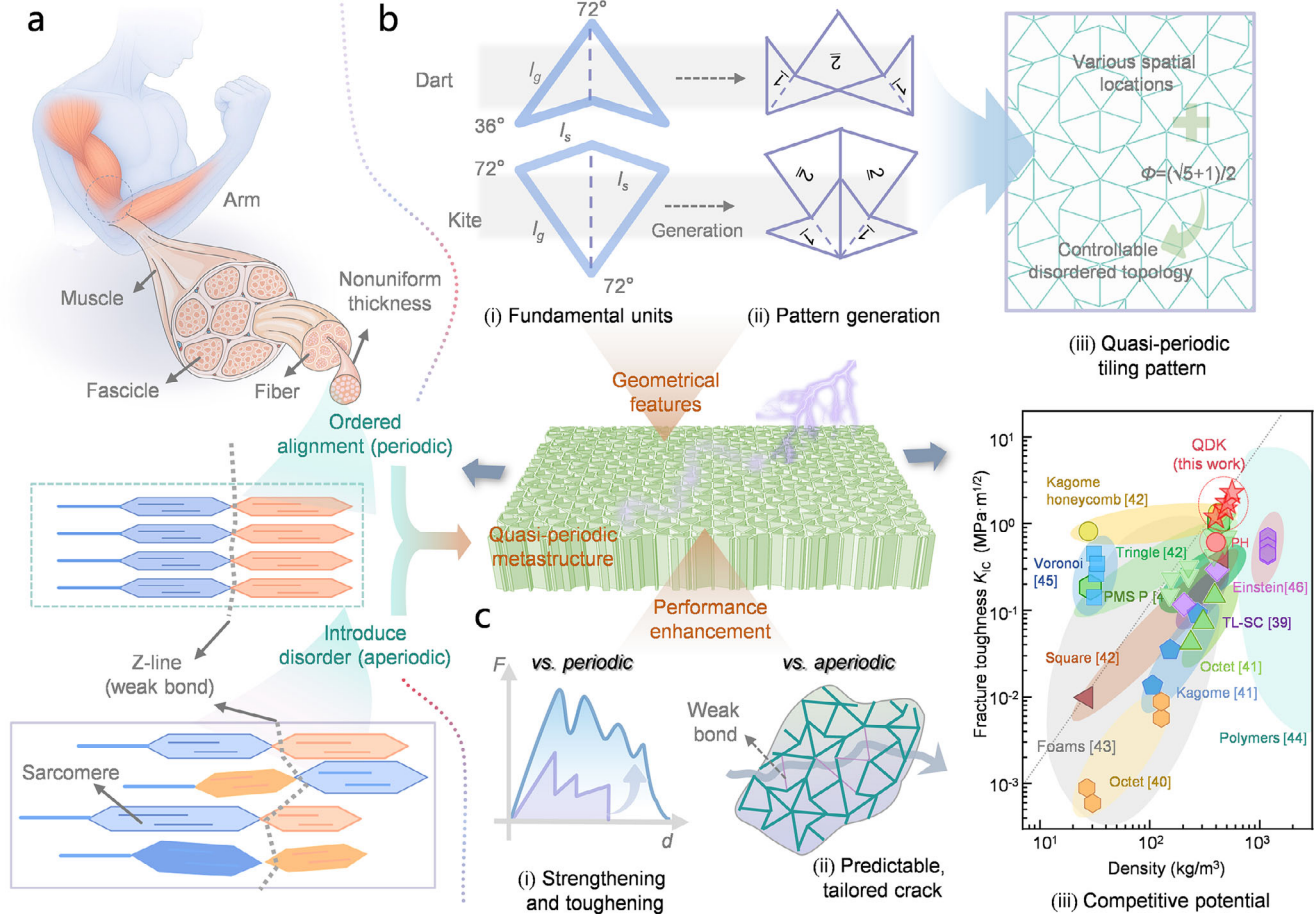
QDK design inspired by the order‐disorder synergy of muscle. (a), Coupling of static‐order with dynamic‐disorder via sacrificial *Z*‐line bonds. (b), Structural geometry construction from Dart‐Kite unit to the macroscopic topology. (c), Concurrent strengthening and toughening arise from the strong‐weak bond network, accompanied by tailored crack propagation [39, 40, 41, 42, 43, 44, 45, 46].

To address the trade‐offs among strength, toughness, geometric controllability, and mechanical predictability, recent research has increasingly drawn inspiration from biological systems, enabled by the geometric design freedom of additive manufacturing [27, 28, 29, 30, 31]. Gradient mineralization in bone and the hierarchical fibrillar architecture of spider silk have inspired gradient and multilevel designs that provide distinct energy‐dissipation and crack‐arresting mechanisms [29, 32, 33]. While these strategies alleviate the strength‐toughness conflict to some extent, gradient structures remain fundamentally aperiodic and hierarchical ones still rely on periodic order, leaving the core challenges of both structural classes unresolved. By contrast, skeletal muscle offers a more instructive model of order‐disorder synergy [34, 35] (Figure 1). (1) At the macroscopic scale, muscle fascicles, fibers and sarcomeres maintain regular alignment and clear load‐transfer pathways (periodic). (2) When external forces are applied, fascicles and fibers exhibit natural variations in diameter, orientation and geometry, which create local freedom for deformation; Sarcomeres, organized periodically at rest, can rapidly elongate and slide relative to each other under high‐rate stretching, producing a transiently disordered state (aperiodic). (3) *Z*‐line emerged in this coupled response functions as a sacrificial interface that absorbs energy and protects the overall structure, allowing muscle to withstand instantaneous tensile forces of several hundred newtons without catastrophic failure (weak bond). Together, these features demonstrate that muscle's mechanical robustness stems from an order‐disorder regime, where order ensures predictable and stable performance, disordered fluctuations dissipate energy and impede crack growth, and weak bands confine damage to predetermined regions. An intriguing question arises: Can engineered topologies replicate this order‐disorder coexistence, using disorder to enhance toughness while geometric constraints preserve structural controllability and mechanical predictability, and enable tailorable failure pathways through coupled mechanisms?

Building on this idea, a pattern constructed from Dart–Kite units is examined. Defined by fixed angles and the golden ratio, this geometry removes translational symmetry while retaining strict local order, yielding a representative quasi‐periodic topology that is aperiodic yet controllable. The resulting thin‐walled quasi‐periodic metastructure (QDK) combines deterministic geometric rules with lightweight structural form, offering distinctive opportunities for mechanical enhancement. Their unit configurations span ten distinct spatial arrangements, creating a significantly expanded design space relative to periodic lattices and imparting inherent advantages for structural performance tuning. Unlike foams or random honeycombs [36, 37], QDKs exhibit no stochastic disorder; unit orientation, edge‐length ratios and tiling sequences are fully dictated by topological determinism. This strict geometric prescription ensures high reproducibility and broad latitude for engineered property modulation. These attributes produce distinct mechanical consequences. Under quasi‐static compression, QDKs form a more heterogeneous and spatially distributed load‐transfer network than periodic lattices, reducing directional sensitivity and improving overall robustness [26]. Relative to disordered structures, they also exhibit more stable deformation modes and higher energy‐absorption capacity [38]. Despite encouraging gains in bulk compressive performance, the mechanisms operating at the failure scale remain largely unresolved. In particular, how quasi‐periodic topology modulates crack initiation, deflection and stress‐field reorganization, and how these processes jointly enable strength, toughness and crack‐path control in lightweight systems, remain poorly understood. A mechanistic understanding that captures these behaviors, as well as design strategies suitable for engineering implementation, is currently lacking.

To this end, two complementary directions are pursued. First, the toughening mechanisms and the strong‐weak bond network inherent in quasi‐periodic topology are identified, and how relative density and global orientation regulate damage evolution in QDK is investigated. Second, to expand application potential, architected weak bonds are introduced into QDK—an architectural analogue of the muscular *Z*‐line—which enable precise crack control while retaining excellent damage tolerance and imposing minimal compromise on global strength and toughness. Moving beyond previous work focused primarily on bulk energy absorption, this work explores the integration of periodic and aperiodic properties with weak‐bond architectures, enabling lightweight protective structures that simultaneously achieve high strength, toughness, exceptional damage tolerance, and tailorable fracture trajectories.

## 2 Results and Discussion

### 2.1 Bio‐Inspired Architecture Toughening With Quasi‐Periodic Strong‐Weak Bonds

Inspired by the natural toughening strategy and tailorable crack pathways observed in muscle tissues, controlled disorder is identified as a key principle for balancing strength, toughness, and both the predictability and tunability of structural performance. Guided by this concept, a quasi‐aperiodic tiling composed of Dart and Kite units is employed as the fundamental geometric motif for constructing the metastructures (Figure 1b). The prescribed characteristic angles and golden‐ratio constraints impart a regulated geometric heterogeneity to the tiling, even though it inherently lacks translational symmetry. The two units possess distinct apex angles, defined by the junction of their long and short edges: 36° for the Dart and 72° for the Kite. A line drawn between the junction of the long sides and the junction of the short sides partitions both units into two congruent isosceles triangles. The classical golden ratio *φ* = (√5+1)/2 is embedded throughout the tiling, governing the number ratio, area ratio, between Kite and Dart units, as well as long‐to‐short edge length ratio (*l_g_*/*l_s_*). Leveraging the recursive generation rules dictated by *φ*, deterministic quasi‐periodic patterns are constructed and further extended into metastructures QDK. Unlike periodic structures, QDK displays natural variations in unit orientations and connectivity, reflected in its accommodation of five characteristic angular relations (36°, 72°, 108°, 144°, and 180°). The detailed procedure for generating the quasi‐periodic tiling is provided in Section S1.

**FIGURE 2:**
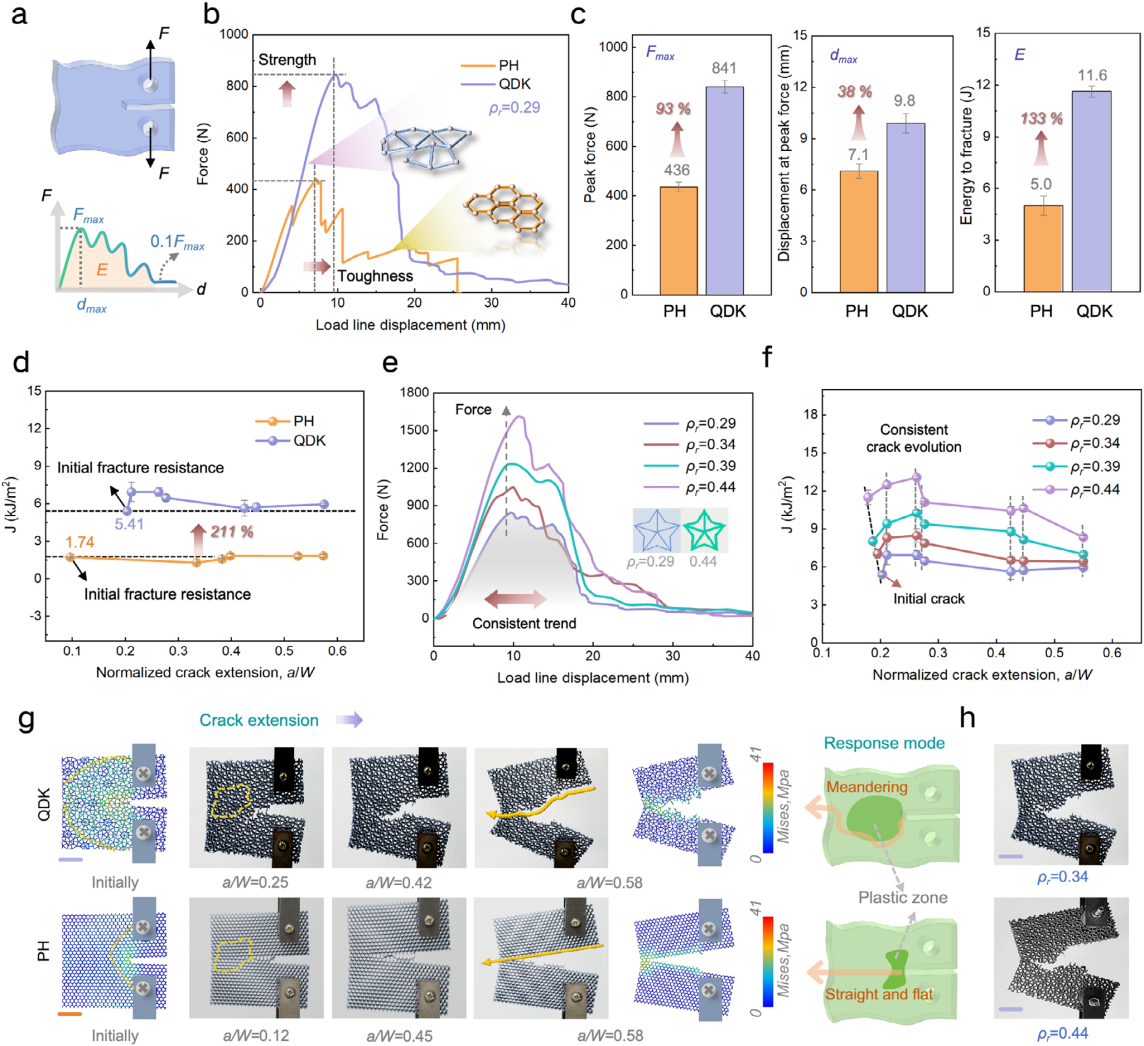
Enhanced mechanical performance of QDK. (a), Loading schematic of single‐edge‐notched specimens and definitions of key mechanical metrics. (b), Force‐displacement curves of PH and QDK. (c), Quantitative comparison of peak force *F_max_*, corresponding displacement *d_max_*, and fracture energy *E* for the two structures. (d), J‐R curves of PH and QDK. (e), Force‐displacement curves of QDKs at relative densities *ρ_r_* = 0.29‐0.44. (f), J‐R curves of QDKs at different relative densities. (g), Experimental and simulated crack‐propagation sequences for PH and QDK. (h), Fractographies of QDKs across different densities. Scale bar: 15 mm.

The fracture behavior of QDK and the periodic honeycomb (PH) is compared to elucidate the toughening mechanisms introduced by quasi‐periodic topology. PH and QDK represent, respectively, a topology with strict translational symmetry and a quasi‐periodic configuration incorporating controlled disorder. All specimens are prepared in accordance with the ASTM E1820 standard [47], with prefabricated cracks introduced during fabrication (Figure 2a). To ensure a fair comparison, the overall dimensions, relative densities, and effective unit resolutions of the two structures are matched so that any mechanical differences arise solely from topology. The number of units included in each specimen is also selected to ensure meaningful structure‐level responses. Details of the structural parameters and equivalence design principles are provided in the Methods and Section S2.

The force‐displacement curves show that QDK achieves superior load‐bearing capacity and fracture toughness, overcoming the typical strength‐toughness trade‐off observed in periodic structures (Figure 2b). PH exhibits a three‐stage response: an initial linear‐elastic regime, a limited nonlinear regime, and a sudden global instability associated with catastrophic crack propagation. After reaching peak load, cracks nucleate and rapidly propagate along periodic weak paths, producing abrupt load drops and a brittle, fully penetrating failure. This behaviour reflects the synchronized amplification of stress concentrations inherent to periodic structures. In contrast, QDK shows a smoother transition from linear response to failure and avoids the abrupt global instability observed in PH, even when load reductions occur. Crack propagation is markedly suppressed, allowing the structure to sustain higher loads over an extended deformation regime. This progressive failure mode stems from the quasi‐periodic topology, where variations in unit geometry, connectivity, and void distribution disrupt coordinated stress amplification and prevent the formation of continuous fracture paths. Overall, the higher load plateau, wider nonlinear regime, and stable damage evolution of QDK indicate that controlled disorder promotes favourable stress redistribution, enabling simultaneous enhancements in strength and toughness in a relatively brittle material system. These observations are further supported by the quantitative metrics in Figure 2c (definitions in Figure 2a). The peak force *F_max_* of QDK increases from 436 N in PH to 841 N, representing a 93% enhancement. This improvement indicates that the quasi‐periodic topology enables a more robust load‐transfer network prior to crack initiation, resulting in substantially higher resistance to fracture. A similar enhancement is observed in the displacement at peak force *d_max_*, which increases by 38% in QDK. The increase in *d_max_* suggests that crack initiation and propagation are markedly delayed, allowing local deformation to be more effectively redistributed throughout the structure. This delay enhances structural toughness and postpones global instability. Due to the combined contributions of these mechanisms, the fracture energy *E* of QDK reaches 11.6 J, compared with 5.0 J for PH, corresponding to a 133% increase.

To quantify the influence of quasi‐periodic topology on suppressing crack growth, the J‐R curves of PH and QDK are compared (Figure 2d). These curves describe the energy‐release evolution during crack growth and reflect the structure's ability to maintain stable fracture. A clear difference appears at the onset of crack initiation: the initial fracture resistance of PH is 1.74 kJ/m^2^, whereas QDK reaches 5.41 kJ/m^2^, representing a 211% increase. This substantial enhancement indicates that the quasi‐periodic topology creates a larger energy‐dissipation zone before appreciable crack‐tip advance, effectively delaying crack initiation. Finite‐element simulations corroborate this trend (Figure 2g), showing a substantially larger initial high‐stress region in QDK. The contrast widens during crack extension. PH maintains nearly constant or slightly decreasing J values, consistent with a straight fracture surface with minimal blunting or arrest. QDK displays a rising J value from the onset of growth, meaning that each increment of crack advance requires higher driving force and that J remains high throughout. These complex crack pathways engage a larger portion of the cellular network, transforming failure from the sudden penetrating mode seen in PH into a gradual fracture evolution. As a result, QDK achieves both high load‐bearing capacity and high crack resistance.

To examine how quasi‐periodic topology behaves across different relative densities, the crack‐propagation responses of QDK with relative density *ρ_r_* = 0.29‐0.44 are compared. As density increases, the load level rises significantly, indicating that load‐bearing capacity can be enhanced through density tuning. However, this improvement does not come at the expense of toughness, in stark contrast to the opposing stiffness‐toughness trend commonly observed in periodic structures [48]. Despite differences in density, all curves of QDK exhibit highly consistent evolution trends (Figure 2e). The J‐R curves further reveal the influence of density on crack resistance (Figure 2f). QDK of different densities all show a rapid early increase in J, followed by a stable high‐resistance plateau, reflecting substantial crack‐tip blunting induced by geometric perturbations in the quasi‐periodic topology. Higher densities not only raise the initial fracture resistance but also maintain higher J values throughout crack growth, indicating enhanced process‐zone energy dissipation. Aside from minor differences in initial crack size, the mid‐to‐late crack sizes are nearly identical across densities, indicating that once stable crack growth begins, the evolution is governed primarily by topology rather than density. This density‐invariant crack‐resistance response highlights the topological adaptivity of QDK.

An instructive phenomenon is observed: QDK of different densities consistently develop nearly identical meandering crack pathways during fracture (Figure 2h). This indicates the presence of an inherently stable strong‐weak bond within the structure, enabling the quasi‐periodic topology to maintain a stable fracture mode across varying densities, as further demonstrated in the following sections. Moreover, compared with other periodic structures (Figure 1c; Section S6), QDK also shows significant improvements in strength and toughness.

### 2.2 Rotating Crack‐Path Maps and Orientation‐Robust Mechanics

**FIGURE 3:**
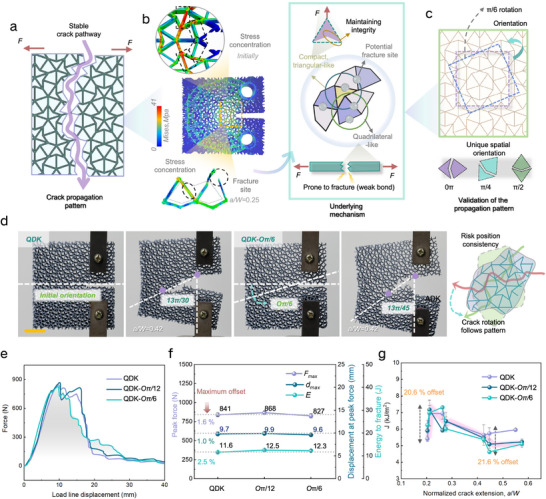
Predictable meandering crack pathways in QDK governed by strong‐weak bonds and their orientation‐robust mechanical response. (a), QDK, unlike aperiodic structures, couples a controllable complex topology with predictable crack pathways. (b), A strong‐weak bond network defined by Dart and Kite units directs crack propagation. (c), Base patterns of QDKs at different orientations. (d), Crack pathways rotate in concert with geometric orientation, demonstrating the robustness of the crack‐shaping mechanism. Orientation‐invariant fracture performance of QDK: (e), force‐displacement curves; (f), mechanical metrics; (g), J‐R curves. Scale bar: 15 mm.

Although some aperiodic structures, such as foam‐like systems, can achieve mechanical performance comparable to QDK (Figure 1c), the predictable fracture behaviour enabled by QDK's controllable, complex topology provides a clear functional advantage (Figure 3a). Across a wide range of relative densities, QDK preserves nearly identical crack pathways, indicating that its strong‐weak bond robustly constrains the failure mode (Section 2.1). The geometry of the Dart and Kite units further maps directly onto the observed crack pathways (Figure 3b). Fractography reveals that the two units differ markedly in stability under load: the compact, triangle‐like Dart unit remains structurally intact, whereas the quadrilateral‐like Kite unit has lower stiffness and tends to concentrate stress, leading to early buckling or fracture. These contrasts give rise to a distinct strong‐weak bond network within QDK. The strong bonds, corresponding to all the short edges of the Dart and Kite units, constitute a stable load‐bearing backbone. By contrast, the weak bonds are primarily located along the long edges of the Dart and Kite units. Due to their higher susceptibility to damage, these weak bonds progressively merge during fracture, ultimately forming a preferential pathway for crack propagation. As a result, the strong‐weak bond network establishes a geometry‐governed crack‐guiding field that allows cracks to propagate along predictable pathways even within a complex topology.

To test the robustness of the crack‐shaping mechanism and assess whether geometric orientation affects the strong‐weak bond network and the resulting crack pathways, global rotations are introduced to the QDK to fabricate specimens with different orientations (Figure 3c). The set comprises the unrotated QDK and configurations rotated by π/12 (QDK‐Oπ/12) and π/6 (QDK‐Oπ/6). The orientation angle is based on the five‐fold symmetry (*n* = 5) of the underlying pattern in QDK, a common approach in anisotropy studies of multicellular structures [49]. The fundamental domain is calculated as *θ* = 2π/*n*, resulting in 2π/5. Rotations beyond this domain cause pattern repetition (equivalent to mirroring), and only configurations within *θ*/2 remain unique. Therefore, the effective rotation domain for QDK is limited to no more than π/5. Furthermore, these small angular shifts enable a sensitive evaluation of the orientation dependence of crack propagation. Fractographies show that the crack pathway in QDK rotates in concert with the structural orientation (Figure 3d). In the unrotated QDK, the crack follows a characteristic meandering pathway that coincides with the preferred failure path defined by the internal strong‐weak bond. After rotating the pattern by π/12 or π/6, the crack direction shifts systematically and remains aligned with the imposed structural orientation. In the π/6 case, for instance, the crack deviates by approximately 13π/90, closely matching the designed orientation, indicating high sensitivity of the crack pathway to the geometric orientation. This behavior arises because the strong‐weak bond network in QDK rotates together with the structure, keeping fracture localized along the long edges of the rotated Kite units (weak bond). Thus, despite the globally quasi‐periodic topology, the strong‐weak bond maintains clear geometric traceability, allowing the crack to follow the same high‐risk regions as the structure rotates. This coherence confirms the crack‐shaping mechanism and underpins targeted crack‐path design (Section 2.3).

The mechanical response of QDK under different geometric orientations is further examined to verify that tunable crack pathways do not compromise its load‐bearing capacity and fracture resistance. The force‐displacement curves for all three orientations exhibit nearly identical progressive behavior, indicating that global orientation has minimal influence on overall strength (Figure 3e). Quantitative metrics reinforce this observation (Figure 3f): the peak load *F_max_*, the corresponding displacement *d_max_*, and the fracture energy *E* vary by only 1.6%‐2.5% across orientations. The J‐R curves further demonstrate robust fracture resistance, with all specimens displaying a similar rising segment at crack initiation followed by a nearly overlapping high‐toughness plateau (Figure 3g). The ∼20% deviation observed in the later stage arises from normal geometric perturbations and does not affect the toughness, load‐bearing capacity, and energy absorption. Collectively, these results show that QDK offers tunable crack pathways while maintaining orientation‐invariant mechanical performance—a key characteristic for robust and tailorable fracture design.

### 2.3 Tailorable Protective Response and Damage Tolerance Mechanism

**FIGURE 4:**
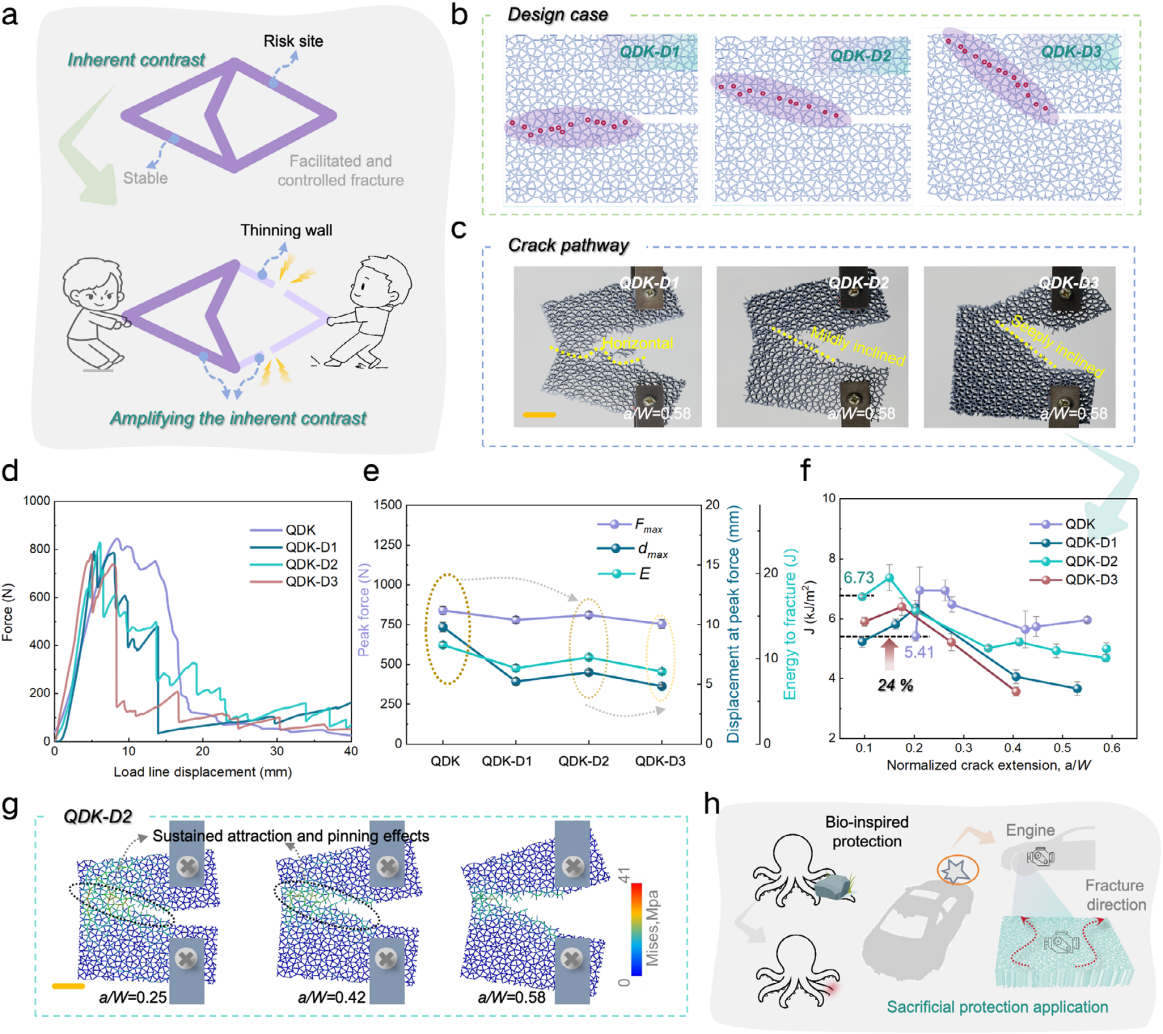
Tailoring crack pathways and their mechanical consequences. (a), Principle of tailoring crack pathways through architected weak bonds. (b), Tailored designs: QDK‐D1, QDK‐D2, and QDK‐D3. (c), Crack‐path evolution in different designs, confirming the effectiveness of the strategy. (d), Force‐displacement curves for the different designs. (e), Mechanical metrics for the different designs. (f), J‐R curves for the different designs. (g), Crack‐propagation sequence of QDK‐D2. (h), Sacrificial protection application. Scale bar: 15 mm.

Adjusting crack direction solely through global rotation of the pattern is insufficient for engineering scenarios that require diverse and tailorable crack pathways. Inspired by the muscle Z‐line, a design strategy is introduced that amplifies the inherent contrast between strong and weak bonds to expand the freedom of crack‐path control (Figure 4a). By geometrically thinning the naturally weak bonds in the Kite units to form architected weak bonds, the local stiffness and strength are deliberately reduced. With the Dart units retained as stable load‐bearing elements, this heightened strong‐weak contrast allows cracks to more readily identify and follow the weakened bonds, thereby initiating and propagating along prescribed pathways. Through targeted weakening of inherently vulnerable regions, crack growth becomes reliably guided toward the desired pathway rather than constrained by the route dictated by the natural topology.

To illustrate the potential of this strategy, three QDK variants featuring distinct induced crack paths are designed: QDK‐D1, QDK‐D2, and QDK‐D3 (Figure 4b). In QDK‐D1, the architected weak bonds are arranged approximately along the horizontal direction. QDK‐D2 introduces weakened weak bonds oriented at roughly π/9 to the horizontal, producing a mildly inclined crack path. QDK‐D3 is based on the rotated QDK‐Oπ/6 configuration, in which weakened weak bonds are placed nearly parallel to π/4, resulting in a large‐angle oblique predefined crack. Together, these three layouts span crack paths from horizontal to multiple inclination angles and account for QDK in different orientations. These cases are representative, and the accessible space for crack‐pathway tailoring is considerably broader than shown here. The strong‐to‐weak bond thickness ratio is 1.7, and additional geometric details of the QDK‐D designs are provided in Section S3.2. Fractographies of the QDK with tailored crack pathways show that cracks in all three designs follow the prescribed directions with high fidelity, confirming that the weakened bonds reliably govern crack propagation (Figure 4c). Numerical results further demonstrate that cracks remain aligned with the weakened bonds, exhibiting a highly stable directional crack pathway (Figure 4g). The locally weakened regions not only define the initiation sites but also generate sustained attraction and pinning during propagation, ensuring that the crack remains locked onto the designed pathway. This effect of control is maintained across QDKs with different orientations, demonstrating that the inductive strategy is robust to geometric variation.

Although crack guidance is introduced by thinning the naturally weak bonds, the QDKs with tailored pathways retain high load‐bearing capacity. The force‐displacement curves show a small and controlled reduction in peak force for QDK‐D1, QDK‐D2, and QDK‐D3 relative to the original QDK (Figure 4d). Notably, QDK‐D3 shows a sudden force drop at a tensile displacement of ∼10 mm. This behaviour arises from the large inclination of its designed crack pathway, which causes the crack to propagate along an extreme direction where fewer edge units are available to engage and redistribute the external load. In addition, the steep pathway results in a shorter crack length, causing the force to decay more rapidly than in the other designs. These results also underscore the performance benefits of a meandering crack, demonstrating the unique advantages of the quasi‐periodic topology. The mechanical metrics further support the above conclusion (Figure 4e). The variations in peak force *F_max_*, corresponding displacement *d_max_*, and fracture energy *E* across different induced pathways remain within a narrow range. Although a slight performance reduction is observed after pathway tailoring, this primarily results from the shorter designed crack paths relative to the natural pathway. Nevertheless, even with these shorter pathways, the structures retain stable energy‐dissipation capacity. Amplifying the strong‐weak contrast markedly increases the initial fracture resistance of the tailored designs while maintaining toughness levels comparable to QDK throughout loading (Figure 4f). QDK‐D2 shows a 24% increase in initial toughness relative to QDK. All three tailored designs exhibit an early rising trend similar to QDK, indicating enhanced resistance to crack initiation. QDK‐D2 attains a higher J, consistent with its longer and more meandering crack pathway (Figure 4g). By contrast, QDK‐D3 displays a slightly lower J with a more pronounced decline in the later stages, attributable to the large inclination of its crack path, which accelerates the reduction in energy dissipation. Tailoring crack pathways is fully compatible with maintaining the mechanical performance of the structure. Details of the theoretical model for predicting the J‐R curve based on the prescribed crack path are provided in Section S7. This architected weak‐bond strategy therefore offers substantial application potential. The self‐sacrificial behaviour of octopus arms provides an instructive analogue (Figure 4h): by prescribing crack pathways, a structure can actively select its fracture pathway under external loading, confining damage to non‐critical regions and protecting the overall system or sensitive components such as precision or high‐value instruments.

To further assess the damage tolerance advantages of quasi‐periodic topologies, cyclic loading tests are performed on the PH, QDK, and QDK‐D3 under both displacement‐controlled and force‐controlled modes. Importantly, to evaluate the topology's capacity to mitigate damage under intentionally weakened local conditions, QDK‐D3—the least mechanically robust variant within the QDK‐D family—is selected as a representative comparison.

**FIGURE 5:**
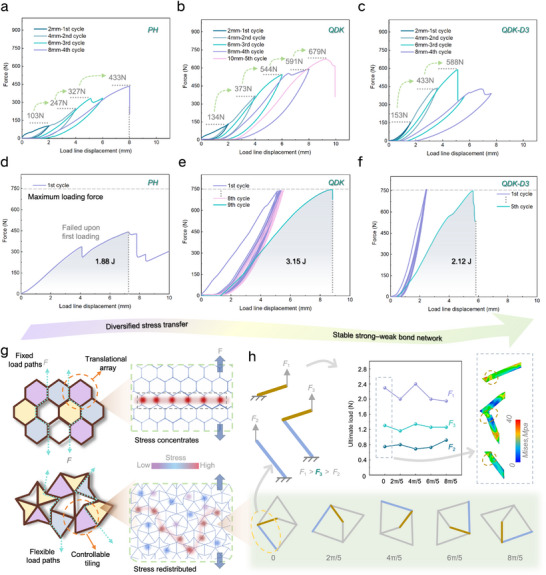
Damage tolerance enabled by the quasi‐periodic topology. (a–c), Force‐displacement curves of PH, QDK, and QDK‐D3 under displacement‐controlled cyclic loading (2–10 mm). (d–f), Force‐displacement curves of the three structures under cyclic loading with a constant peak load of 750 N. (g,h), Quasi‐periodic topology with a stable strong‐weak bond network reshapes stress distribution, contributing to competitive mechanical performance compared with periodic and aperiodic structures.

The displacement‐controlled cyclic responses of the three structures reveal clear performance distinctions, with the quasi‐periodic topology showing superior damage suppression and energy dissipation. For PH (Figure 5a), peak forces remain low across cycles (103 N–327 N), reflecting limited energy absorption. At an 8 mm displacement amplitude, internal damage rapidly coalesces and triggers a catastrophic fracture due to the lack of effective damage isolation. QDK exhibits markedly higher damage tolerance (Figure 5b). Its peak force increases steadily from 134 to 591 N as displacement rises from 2 to 8 mm (∼36% higher than PH at 8 mm). Its cyclic response also remains stable, with fracture occurring only in the fifth 10 mm cycle. Crack growth is consistently fragmented and strongly impeded. QDK‐D3 shows that introducing architected weak bonds shifts crack initiation but does not compromise overall damage controllability (Figure 5c). Its energy dissipation level is comparable to QDK. The localized fracture observed in the third 6 mm cycle results from stress focusing at the weak bonds, initiating cracks earlier along the prescribed pathway. Even so, QDK‐D3 sustains higher peak forces than QDK—by ∼8% in the third cycle—and maintains stable, path‐confined dissipation behavior, with failure strictly limited to the designed trajectory. The force‐controlled results at a constant maximum load of 750 N further reinforce the above trend. PH fractures in the first cycle, absorbing only 1.88 J, confirming its inability to sustain stable dissipation under high loads (Figure 5d). QDK maintains a stable response across repeated cycles (Figure 5e). Its load‐bearing capacity remains well preserved through eight cycles, and failure occurs only in the ninth, where the absorbed energy reaches 3.15 J—nearly twice that of PH. Despite local weakening, QDK‐D3 still outperforms PH (Figure 5f). It remains intact for four cycles at 750 N, and its final fracture absorbs 2.12 J. Its notably smaller hysteresis loop compared with QDK indicates that deformation is captured earlier by the architected weak bonds, thereby limiting distributed local deformation and reducing additional energy dissipation during cycling.

The diversified stress distribution in quasi‐periodic topologies contributes to improved strength, toughness, and damage tolerance (Figure 5g). In periodic structures, translational symmetry confines load transfer to a fixed path, promoting stress concentration and rapid crack propagation along the weakened direction. As a result, failure tends to be brittle, with limited damage tolerance and energy absorption. By contrast, the quasi‐periodic topology breaks strict translational order and forms a multidirectional load‐transfer network. The varying unit orientations and connections redistribute external loads, generating a more dispersed stress field. Under these conditions, cracks are more likely to deflect, branch, and arrest, producing meandering crack paths that reduce the fracture driving force and delay global failure. This distributed damage mode activates more structural units during deformation, thereby improving ductility and energy absorption.

The strong‐weak bond network promotes crack deflection and enables tailored crack paths. To elucidate its formation mechanism, representative truss units are extracted from the QDK for analysis (Figure 5h). Owing to the fivefold symmetry of the pattern, all five spatial orientations of the dart and kite units are considered. The fracture loads of isolated strong bonds, isolated weak bonds, and coupled strong‐weak bonds are denoted as *F*_1_, *F*_2_, and *F*_3_, respectively. *F*_1_ remains markedly higher than *F*_2_ across all spatial angles, indicating a clear fracture‐limit contrast between strong and weak bonds. Consequently, weak bonds fracture first and govern crack deflection. When both coexist, *F*_3_ is slightly higher than *F*_2_ but remains far below *F*_1_, indicating that weak bonds still dominate fracture initiation despite the coupling effect. The corresponding response contours further confirm the robustness of the strong‐weak bond network. On this basis, architected weak bonds convert topology‐driven crack deflection into an adjustable fracture trajectory. By capturing cracks at an early stage, they direct damage along the prescribed path. Despite earlier crack initiation within the induced band, the intrinsic stress dispersion of the quasi‐periodic topology still governs the overall response, allowing QDK‐D to achieve path‐controlled fracture while retaining high damage tolerance and energy absorption.

## 3 Conclusion

A deterministic quasi‐periodic QDK metastructure is introduced, integrating high fracture resistance, damage tolerance, and mechanical programmability within a single lightweight architecture. Quasi‐periodicity eliminates translational symmetry without abandoning rule‐based design, reshaping how stresses percolate through the network and how damage organizes once a crack is activated.

In tension, QDK replaces the synchronized, weak‐link rupture typical of periodic patterns with a distributed, process‐zone‐dominated failure sequence. Stress is re‐routed away from a single repeating bottleneck; plastic deformation spreads, then stabilizes crack growth through deflection, branching, and arrest. Quantitatively, relative to a density‐matched periodic honeycomb, QDK achieves a 211% increase in initial fracture resistance, 93% higher peak force, 38% greater peak displacement, and a 133% gain in fracture energy. Under cyclic loading, the response remains stable, with energy absorption during the fracture cycle reaching 3.15 J, approximately 68% higher than that of the periodic reference (1.88 J). Load‐bearing capacity is further tuned via relative density without compromising toughness.

Fracture control emerges as a built‐in function rather than a byproduct of randomness. An intrinsic strong‐weak bond network encodes a topology‐governed crack map that rotates deterministically with architecture orientation, while strength and toughness remain essentially invariant (<2.5% variation). Z‐line‐inspired weak bands extend this principle to on‐demand crack routing—horizontal, mildly inclined, or steep—while preserving global performance; notably, QDK‐D2 even raises initial fracture resistance by 24%. Collectively, QDK elevates quasi‐periodicity into a practical design principle for lightweight structures that must be both tunable and reliably tough, with failure modes engineered as part of the architecture.

## 4 Materials and Methods

### 4.1 Fabrication of Quasi‐Periodic Matastructures

Quasi‐periodic tilings composed of Dart and Kite units were first generated in PyCharm and MATLAB, where preliminary 2D layouts were produced from stochastic seed patterns. The resulting dxf sketches were imported into SolidWorks (Dassault Systèmes, USA) for extrusion and Boolean operations, and subsequently exported as STereoLithography (STL) files for 3D printing. Polylactic acid (PLA, density 1.23 × 10^3^ kg/m^3^) was selected for its biodegradability and excellent compatibility with established additive manufacturing workflows. Printing was performed at 200–220°C with a 0.4 mm nozzle, 0.2 mm layer height, an infill speed of 200 mm/s, and a 99% wall‐free infill adopting a 3D hexagonal pattern. Representative metastructures fabricated under these parameters are shown in Figure 2. Relative densities of 29.33%, 34.39%, 39.47%, and 44.17% were achieved (corresponding to wall thicknesses of 0.5–0.8 mm). A periodic honeycomb structure of equivalent density (29.33%; 0.607 mm wall thickness) served as a control. All specimens were printed with a consistent orientation along the principal stretching direction of the pattern, and the geometric fidelity, deviations, and effects of alternative orientations on performance are discussed in Section S3.

### 4.2 Mechanical Testing

PH and QDK followed the geometric scaling of ASTM E1820 compact‐tension (CT) specimens [47] (Figure S3). Given the absence of explicit standards for fracture testing of cellular structures, the common guideline that a larger number of cells yields more representative failure behavior was adopted. Previous studies indicated that approximately 400 in‐plane cells were required for convergence in periodic structures [50]. By equivalence and considering the quasi‐periodic structure used here, more than 500 in‐plane cells were used to ensure representative fracture responses. Fracture tests were conducted on a universal testing machine (Z020, ZwickRoell GmbH, Germany) equipped with a 5 kN load cell. Tensile loading proceeded at 2.0 mm/min. Cyclic loading used symmetric loading/unloading rates of 2.0 mm/min with displacement amplitudes of 2, 4, 6 mm and so forth, or under a constant maximum load of 750 N. Crack evolution was recorded using a Canon EOS 5D Mark IV camera (6720×4480 pixels; ∼0.1l_0_ × 0.1l_0_ per pixel). Synchronous force‐displacement and imaging data were used to extract fracture toughness and quantify crack‐growth behavior. Fracture toughness was calculated using the J‐integral method, following the procedure outlined by ASTM, with details outlined in Section S4. Although ASTM standards are intended for continuous materials, many studies still use this method for architected materials in the absence of universally accepted alternatives [43, 51] The apparent/effective toughness from ASTM can provide meaningful comparisons between different design configurations. All experimental results were derived from two identical independent trials, with the results averaged [52, 53]. A third trial would be conducted only if significant discrepancies were observed. Since the results were consistent, no third trial was necessary.

### 4.3 Finite Element Simulation

Finite‐element (FE) simulations were carried out using Abaqus/CAE 2022 (Dassault Systèmes Simulia, USA), reproducing the experimental geometries and loading conditions (Figure S8). Because failure in these thin‐walled structures occurs predominantly in‐plane under quasi‐2.5D tensile loading, the models employed a 2D‐dominant meshing strategy for computational efficiency. An element mixture analogous to 8‐node linear brick elements with reduced integration (C3D8R) and 6‐node triangular prism elements (C3D6) was used to maintain geometric fidelity near intricate features. Mesh sizes ranged from 0.2–0.3 mm, resulting in approximately 3–5 million elements per model. PLA was modelled as an elastic‐plastic material, with constitutive parameters obtained from tensile tests following ASTM D638 [54]. The elastic regime was described using a Young's modulus of 2.93 GPa and a Poisson's ratio of 0.32. Plasticity was characterised using the true stress‐plastic strain relation directly extracted from experiments (Figure S7). Fracture was simulated via element deletion enabled by a damage model with a plastic strain at failure of 0.11. A linear damage evolution law governed by a fracture energy of 4 kJ/m^2^ was applied [43].

## Conflicts of Interest

The authors declare no conflict of interest.

## Supporting information




**Supporting File**: advs75413‐sup‐0001‐SuppMat.docx.

## Data Availability

The data that support the findings of this study are available from the corresponding author upon reasonable request.
