# Supplementary material for: Topology‐Enriched Toughness Enhancement in Quasi‐Periodic Metastructures Featuring Tailorable Strong‐Weak Network
Source: Adv Sci (Weinh). 2026 Apr 20;13(40):e75413. doi: 10.1002/advs.75413 (PMC13335715; doi:10.1002/advs.75413)
Supplement: Supplementary file 1 — Supporting File: advs75413‐sup‐0001‐SuppMat.docx. [file ADVS-13-e75413-s001.docx]

Supplementary materials

Topology-enriched toughness enhancement in quasi-periodic metastructures featuring tailorable strong-weak network

Tianyu Gao ^a^, Genda Wang ^b^ *, Jiabao Bai ^a^, Kai Liu ^c^ *

^a^ Department of Engineering Mechanics, Zhejiang University, Hangzhou, Zhejiang, China

^b^ Institute of Advanced Equipment, College of Energy Engineering, Zhejiang University, Hangzhou, Zhejiang, China

^c^ Frontiers Science Center for Extreme Flows and Energies, School of Traffic & Transportation engineering, Central South University, Changsha, Hunan, China.

* Corresponding authors: [g.wang@zju.edu.cn](mailto:g.wang@zju.edu.cn) (G. Wang), [liu_kai@csu.edu.cn](mailto:liu_kai@csu.edu.cn) (K. Liu)

This Supporting Information provides further details of the main text and is organized as follows.

- - Section S1: Generation of quasi-periodic tiling pattern
- S1.1 Geometry of fundamental units
- S1.2 Pattern generation strategy
  - Section S2: Equivalent pattern resolution
  - Section S3: PH and QDK specimens
- S3.1 Geometry of the compact-tension (CT) specimen
- S3.2 Printing accuracy analysis
- S3.3 Effect of printing orientation on the fracture response of QDK
  - Section S4: Fracture toughness calculation
  - Section S5: Material characterization and finite element model validation
- S5.1 Tensile testing
- S5.2 Finite element model validation
  - Section S6: Comparison of fracture behaviour between quasi-periodic and typical cellular structures
  - Section S7: Theoretical framework for linking prescribed fracture paths to J-R curves in QDK

Section S1: Generation of quasi-periodic tiling pattern

S1.1 Geometry of fundamental units

The quasi-periodic tiling composed of Dart and Kite units represents a canonical Penrose tessellation (**Figure S 1**). Using only two geometric primitives, the pattern achieves seamless coverage of an infinite plane [1]. Unlike periodic tessellations, it lacks translational symmetry at any scale. In contrast to stochastic aperiodic arrangements, however, the pattern is tightly constrained by deterministic matching rules, producing a characteristic form of controlled disorder. Owing to this combination of structural determinism and aperiodicity, Penrose tilings have been extensively examined in the design of functional mechanical metamaterials [2].


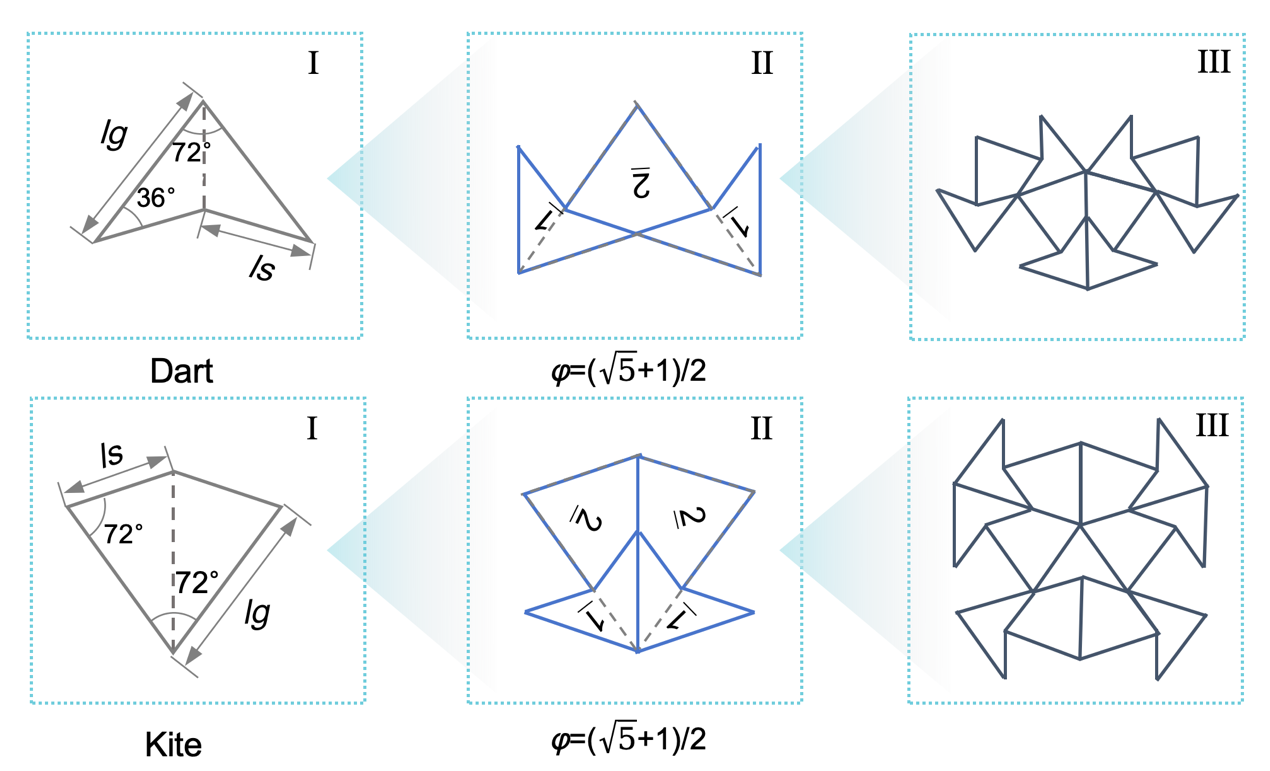


**Figure S 1.** Geometric characteristics and tiling process of the quasi-periodic pattern composed of Dart and Kite units.

The Dart and Kite share identical long and short edges (*l_g_*, *l_s_*) but differ in their interior angles (36° for the Dart and 72° for the Kite). Connecting the endpoints of the long and short edges partitions each unit into two congruent isosceles triangles, providing the geometric basis for substitution rules. A defining attribute of the Penrose tiling is its intrinsic coupling to the golden ratio *φ* (Equation S1). The area ratio, number ratio, and long-to-short edge ratio of the tiles all converge to *φ*, imposing the geometric constraints required for fivefold rotational symmetry and local matching compatibility.

| $\varphi=\frac{S_{k}}{S_{d}}=\frac{N_{k}}{N_{d}}=\frac{l_{g}}{l_{s}}=\frac{\sqrt{5}+1}{2}\approx1.618$ | (S1) |
| --- | --- |

Here, *S_d_* and *S_k_* denote the areas of Dart and Kite; *N_d_* and *N_k_* represent their respective populations. The tiling also exhibits self-similarity, with higher-order patterns formed through uniform inflation of lower-order units by *φ*. This scaling hierarchy preserves geometric ratios across levels, imparting strong scale invariance to an otherwise visually intricate pattern.

S1.2 Pattern generation strategy

The absence of translational symmetry precludes the construction of quasi-periodic patterns using conventional unit array approaches. Baake et al. [3] identified two foundational strategies that provide a systematic framework for generating quasi-periodic structures:

(1) Cut-and-project method

A periodic lattice in higher-dimensional space is intersected by an appropriately oriented slice, which is then projected onto a two-dimensional plane. This operation yields structures that are quasi-periodic yet retain rotational symmetry. The approach elucidates the higher-dimensional geometric origin of quasi-periodicity and underpins theoretical descriptions of a wide range of quasi-periodic systems.

(2) Substitution rule

In this recursive scheme, tiles are subdivided according to prescribed edge-angle constraints and adjacency rules. Each generation is produced by uniform inflation using a fixed scaling factor (**Figure S 1**), thereby maintaining geometric consistency and connectivity across hierarchical levels (first generation, second generation, etc.). In this work, the Penrose tessellation is constructed using a golden-ratio-based substitution rule, which mitigates geometric frustration and ensures conflict-free hierarchical growth.

Section S2: Equivalent pattern resolution

In the design of multi-cellular structures, achieving sufficient decoupling between the macroscopic structural response and the characteristic size of the microstructural units is essential for ensuring stable mechanical performance [4]. Previous studies have shown that, in typical periodic honeycomb topologies, approximately 400 in-plane units are required for the multi-scale effects to fully decouple and for the mechanical properties to converge [5]. Building on this criterion, the present study increases the in-plane unit count to ~500 to establish a more robust scale-separation condition.

To ensure a fair comparison between periodic honeycomb (PH) and quasi-periodic metastructure with Dart and Kite units (QDK), the resolution of both structures is carefully calibrated to be equivalent (**Figure S 2**). Specifically, the long and short edges (*l_g_*, *l_s_*) of the Dart and Kite units, as well as the honeycomb edge length (*lh*), are adjusted such that the area of the honeycomb unit matches the weighted average area of the two units in QDK [6]. This area-based calibration ensures that both topologies operate at comparable spatial resolution. Leveraging the self-similarity inherent to quasi-periodic structures, the equivalent pattern resolution is then determined using:

| $S_{h}=\frac{{\varphi S}_{k}+S_{d}}{\varphi+1}$ | (S2) |
| --- | --- |

where *S_h_* denotes the area of the honeycomb unit.


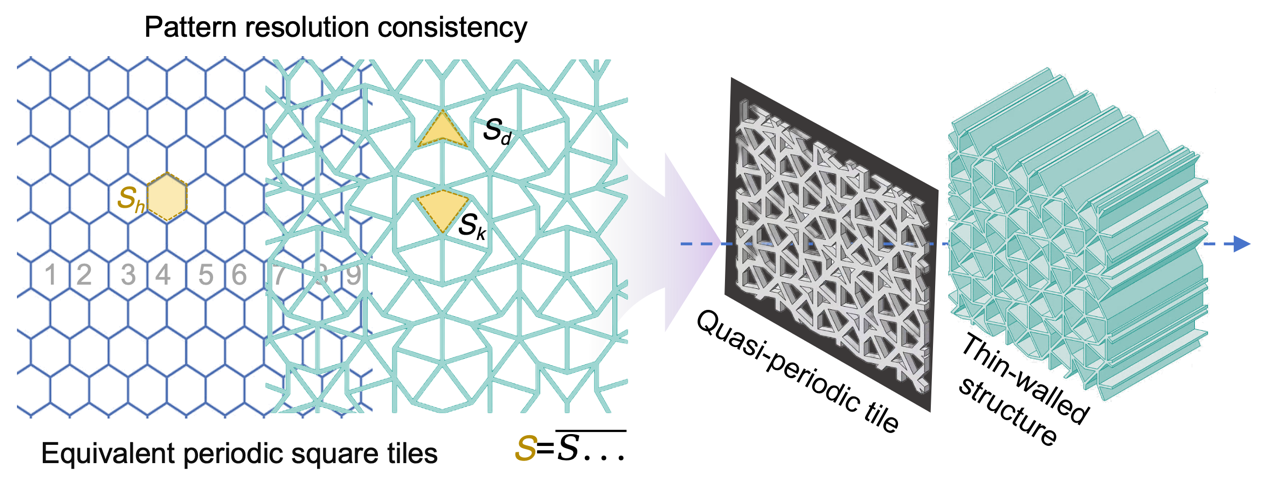


**Figure S 2.** Equivalent resolution of the PH and QDK.

To achieve a moderate level of energy absorption, the edge length *lh* and wall thickness *th* of the reference honeycomb are set to 2.2 mm and 0.607 mm, respectively. Based on the area-equivalence condition, the long edges *l_g_* of the Dart and Kite units in QDK are set to 5 mm. The relative density of PH is calculated as:

| $\rho_{h}=\frac{2t_{h}}{\sqrt{3}l_{h}}-\frac{{t_{h}}^{2}}{3{l_{h}}^{2}}$ | (S3) |
| --- | --- |

The resulting relative density of PH is 29.33%. The wall thickness of QDK is adjusted to 0.5 mm in the CAD model to ensure an identical relative density. This design calibration ensures consistent pattern resolution and relative density across the different topologies, enabling fair mechanical comparisons in Section 2.1 of the main text.

Section S3: PH and QDK specimens

S3.1 Geometry of the compact-tension (CT) specimen

PH and QDK specimens follow the geometry prescribed for compact-tension (CT) specimens in ASTM E1820 [7] (**Figure S 3**). The specimen width is set to *W* = 80 mm, with a thickness of *B* = 20 mm and an overall height of *H* = 96 mm. For both PH and QDK, the equivalent characteristic length of unit edge is defined as *l* = 2.2 mm, resulting in *W* ≈ 36*l* and *B* ≈ 9*l.* This configuration ensures a sufficient separation of macroscopic structural response and the characteristic size of the microstructural units, enabling well-resolved structural fracture responses (see Section S2). A pre-crack of length *a_0_* ≈ 17*l* = 37 mm and height 4 mm is introduced directly during fabrication. The resulting crack-length ratio *a_0_*/*W* ≈ 0.46 lies within the ASTM E1820 recommended range of 0.45-0.55, providing an adequate remaining ligament for stable crack growth and ensuring a well-defined initial crack front.


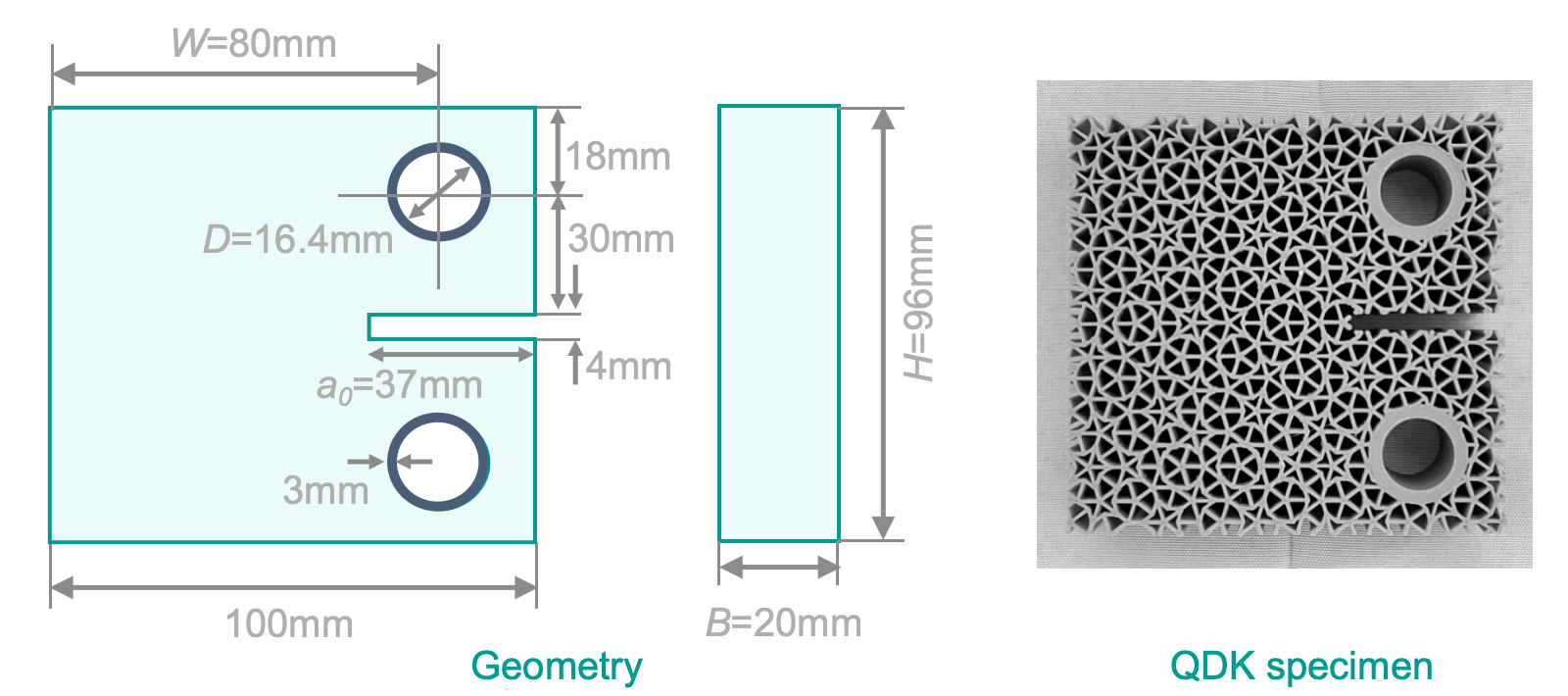


**Figure S 3.** CT-specimen geometry and fabricated QDK specimen.

Because cellular structures such as PH and QDK are prone to localized crushing near the loading boundaries, a 3-mm-thick solid reinforcement ring is added around the loading-pin holes [8]. This reinforcement does not modify the global CT geometry or the intended crack path but enhances the local bearing capacity, ensuring that fracture initiates from the pre-crack rather than from undesired damage near the grips. This design preserves identical boundary conditions for the two structures, ensuring that any observed differences in fracture behaviour arise from their underlying topology rather than from discrepancies in loading or support conditions.

S3.2 Printing accuracy analysis

All specimens are fabricated using fused deposition modeling (FDM), and the printed geometries exhibit close agreement with the corresponding design models (**Figure S 4a**). PH and QDK with uniform wall thickness, as well as the QDK-D1 structure incorporating intentionally varied local wall thickness, all demonstrate high geometric fidelity, with well-defined unit-level features (**Figure S 4b**).

**
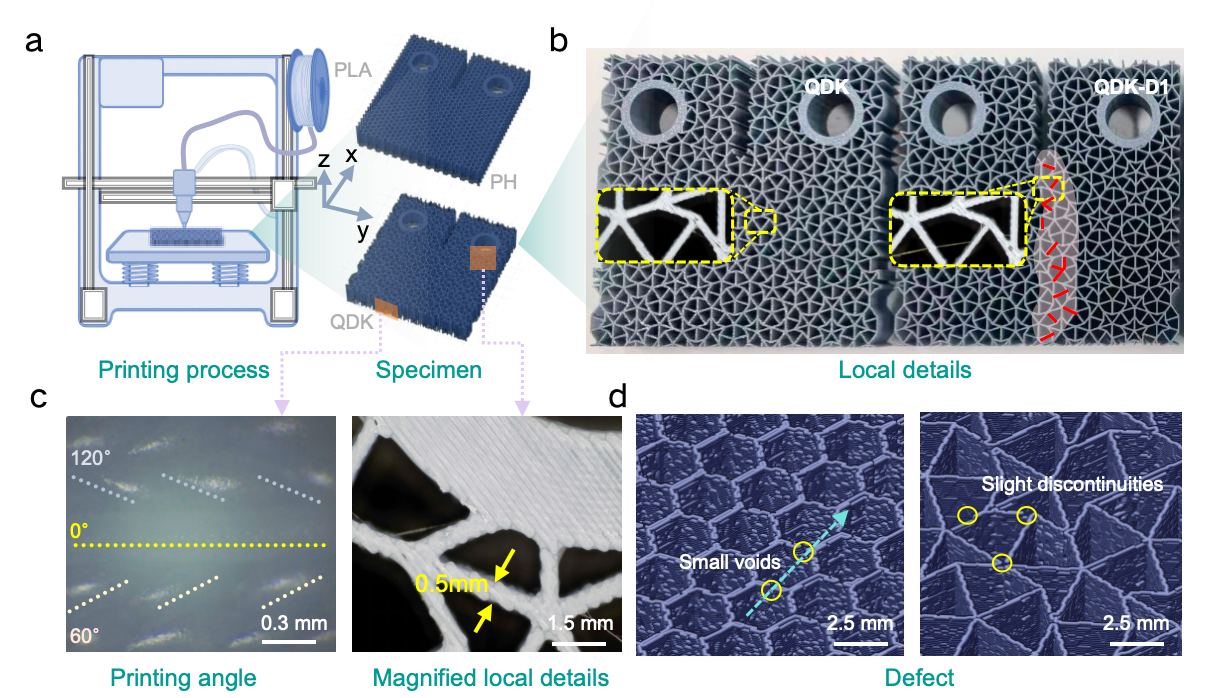
**

**Figure S 4**. Printing accuracy of the fabricated specimens. **a,** Schematic of the fused deposition modeling (FDM) process and representative PH and QDK specimens. **b,** Printed QDK specimen showing uniform wall thickness, and printed QDK-D1 specimen with locally varied wall thickness. **c,** Magnified views along the thickness direction and local wall-thickness details. **d,** Typical printing defects, including small voids and slight discontinuities.

To assess the layer-by-layer deposition characteristics, magnified views along the thickness direction are examined. Distinct extrusion orientations at 0°, 60°, and 120° are clearly resolved, and these periodic deposition paths collectively form a regular hexagonal infill pattern. The measured wall thickness of QDK (0.5 mm) closely matches the prescribed value, confirming that the printing resolution meets the design requirements (**Figure S 4c**). Minor printing defects—such as small voids or slight discontinuities—are occasionally observed (**Figure S 4d**). These defects are limited in scale, thereby exerting negligible influence on the global geometry or on the reliability of mechanical testing [9]. Moreover, comparison of the measured and theoretical masses indicates that deviations for both PH and QDK specimens remain within 10% [10] (**Table S 1**), demonstrating good dimensional consistency and process repeatability.

Taken together, the combined qualitative and quantitative evidence verifies that the FDM process achieves sufficient printing accuracy to support rigorous mechanical comparisons among the different topologies investigated in this study.

**Table S 1**. Mass information of the printed CT specimens.

| Specimen ID | Mass (g)-Specimen | Mass (g)-CAD | Error (%) |
| --- | --- | --- | --- |
| PH | 73.15 | 72.15 | 1.4% |
| QDK | 71.10 | 72.15 | -1.5% |

S3.3 Effect of printing orientation on the fracture response of QDK

To reveal the intrinsic fracture mechanism of the quasi-periodic structures, all mechanical tests in this work are conducted using the printing strategy described in the Methods section, namely, printing along the principal stretching direction of the pattern, corresponding to the *z* direction in the **Figure S 4a**. For thin-walled structures, this orientation is generally more favorable for ensuring geometric accuracy and mechanical performance [11]. Accordingly, the following fracture analysis is based on specimens printed along the *z* direction to minimize the influence of orientation-induced manufacturing deviations.

Nevertheless, printing orientation still strongly affects the forming quality and failure response of the structures. As shown in **Figure S 5a**, specimens printed along the *x* direction exhibit obvious local insufficient fusion, indicating reduced compatibility between the deposition path and the thin-walled geometry, and thus lower geometric fidelity. In addition, the actual printed mass is 64.70 g, lower than the designed mass of 72.15 g, corresponding to a deviation of approximately 10.3%, suggesting that printing-induced mass deviation cannot be neglected when evaluating structural performance [12]. Correspondingly, **Figure S 5b** shows that the peak load of the x-printed specimens is markedly lower than that of the *z*-printed specimens, with a peak-force reduction of approximately 69%, and an earlier onset of instability and softening. The fracture morphology further shows pronounced interlayer delamination during loading (**Figure S 5c**), indicating that the unfavorable printing orientation weakens interlayer integrity and promotes crack propagation along the interfaces.

These results show that printing orientation affects not only the geometric accuracy of quasi-periodic structures, but also their load-transfer behaviour. Consistent with previous studies on thin-walled structures [11, 12], the *z*-direction printing strategy is therefore adopted throughout this work to ensure that the observed fracture behaviour primarily reflects the intrinsic mechanical response of the structure.


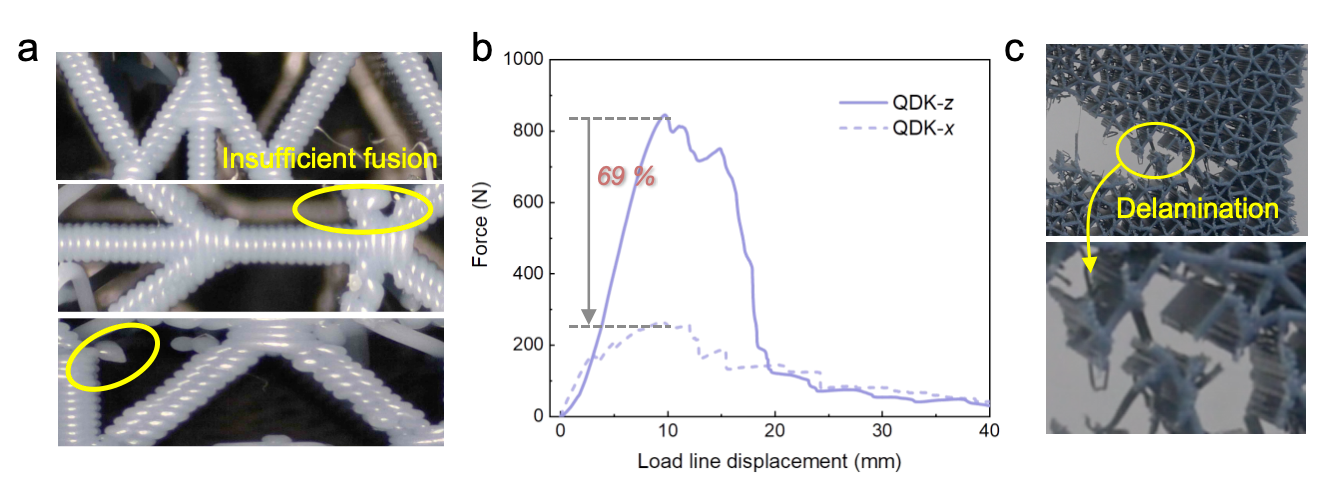


**Figure S 5.** Forming quality and fracture performance of specimens printed in different orientations. **a,** Forming quality of the specimen printed along the *x* direction. **b,** Force-displacement curves of specimens printed along the *x* and *z* directions. **c,** Fractography of the specimen printed along the *x* direction.

Section S4: Fracture toughness calculation

Fracture toughness of the honeycomb and QDK is evaluated using standard compact-tension (CT) specimens and a **J-integral-based analysis procedure (following ASTM E1820 [7])**. During the fracture tests, **load-displacement (*F*-*δ*) data are continuously recorded as the pre-crack extends in a stable manner**. At each evaluation step *i*, the current crack length *a_i_* is **extracted from high-resolution video recordings** (with one-half-unit cell resolution), and the J-integral is expressed as the sum of elastic and plastic contributions:

| $J_{i}= J_{i}^{E}+ J_{i}^{P}$ | (S4) |
| --- | --- |

The elastic component $J_{i}^{E}$ is obtained from the stress intensity factor $K_{i}$ and the **in-plane elastic modulus** *E* of the porous structure:

| $J_{i}^{E}=\frac{K_{i}^{2}}{E}$ | (S5) |
| --- | --- |

where *E* of QDK is calculated using $E=mE_{s}\rho_{r}^{n}$ (with *m* and *n* provided in **Figure S 6**). Owing to the quasi-isotropic elastic and plastic responses of QDK [6, 13], we assessed the stiffness of QDK specimens with varying relative densities under a defined loading orientation to determine the values of *m* and *n.* The instantaneous stress intensity factor $K_{i}$ corresponding to force $F_{i}$ is then obtained as:

| $K_{i}=\frac{F_{i}}{B\sqrt{W}} f\left( \frac{a_{i}}{W} \right)$ | (S6) |
| --- | --- |

with

| $f\left( \frac{a_{i}}{W} \right)=\frac{\left( 2+\frac{a_{i}}{W} \right)}{\left( 1-\frac{a_{i}}{W} \right)^{1.5}}\left[ 0.886+4.64 \left( \frac{a_{i}}{W} \right)-13.32 \left( \frac{a_{i}}{W} \right)^{2}+14.72 \left( \frac{a_{i}}{W} \right)^{3}-5.6 \left( \frac{a_{i}}{W} \right)^{4} \right]$ | (S7) |
| --- | --- |

where $W$ is the specimen width, $B$ is the thickness, and $f\left( a_{i}/W \right)$ is the the **standard geometric calibration function for CT specimens**. To quantify the initial fracture toughness, *K_IC_* is defined as the critical stress-intensity factor at crack initiation under plane-strain conditions.


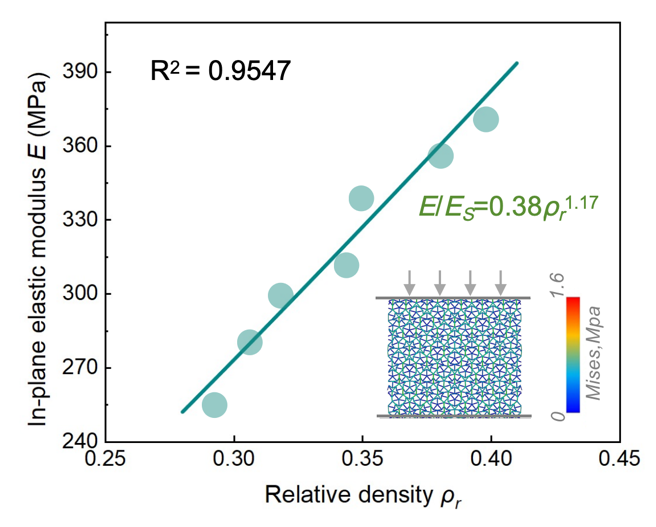


**Figure S 6. In-plane modulus of QDK.**

The plastic component *J_i_^P^* is computed from the **incremental plastic work** obtained from the load-displacement curves:

| $J_{i}^{P}=\left[ J_{i-1}^{P}+\left( \frac{\eta_{i-1}^{P}}{b_{i-1}} \right)\frac{A_{i}^{P}-A_{i-1}^{P}}{B} \right]\left[ 1-\gamma_{i-1}^{P}\left( \frac{a_{i}-a_{i-1}}{b_{i-1}} \right) \right]$ | (S8) |
| --- | --- |

where $A_{i}^{P}-A_{i-1}^{P}$ represents the **incremental plastic area under the *F*-*δ* curve** between step $i-1$ and $i$. The term $b_{i-1}=W-a_{i-1}$denotes the uncracked ligament length, and the geometry-dependent factors are given by

| $\eta_{i-1}^{P}=2+0.522\frac{b_{i-1}}{W}$ | (S9) |
| --- | --- |
| $\gamma_{i-1}^{P}=1+0.76\frac{b_{i-1}}{W}$*.* | (S10) |

Section S5: Material characterization and finite element model validation

S5.1 Tensile testing

The mechanical properties of polylactic acid (PLA) are evaluated through uniaxial tensile tests on dog-bone specimens (following ASTM D638 [14]), with dimensions shown in **Figure S 7a** and **Figure S 7b**. Tests are conducted on a universal testing machine (Z020, ZwickRoell GmbH, Germany) at a constant loading rate of 2 mm/min. Stress-strain responses are recorded throughout the loading process. Five replicate specimens are tested, demonstrating high repeatability and consistent failure behaviour. Fracture morphologies and the corresponding stress-strain curves are presented in **Figure S 7**c and **Figure S 7d**. From these curves, Young’s modulus, Poisson’s ratio, and plastic fracture strain are extracted, and the measured properties are found to show close agreement with the supplier’s reference data, as reported in the Methods section of the main text.


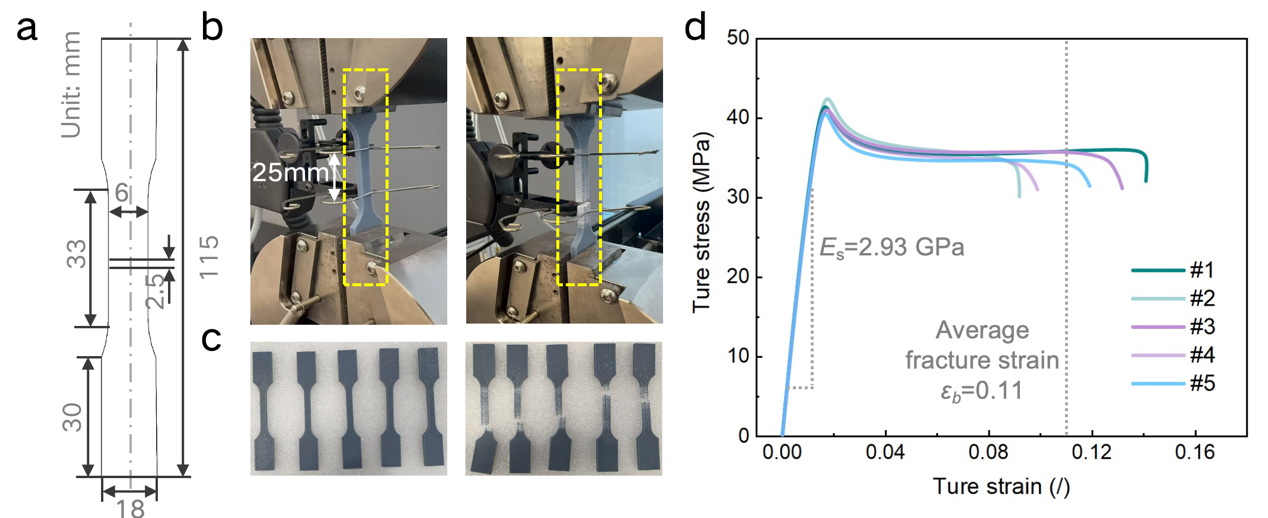


**Figure S 7.** Mechanical characterization of PLA. **a-b,** Dimensions of the dog-bone tensile specimen (unit: mm). **c,** Fracture morphologies observed after tensile testing. **d,** Stress-strain curves of five replicate specimens.

S5.2 Finite element model validation

Finite element simulations are employed to analyze crack initiation and propagation in the quasi-periodic structures. The modelling framework is validated by comparison with experimental results obtained from QDK specimens. All simulations are conducted using the same loading rate of 2 mm/min as in the experiments, ensuring direct comparability (**Figure S 8**). As shown in **Figure S 9a,** the simulated force-displacement responses exhibit strong agreement with the experimental measurements. The crack-evolution sequences in **Figure S 9b** further confirm that the model accurately captures the onset, growth, and trajectory of the fracture process. Overall, these results demonstrate that the finite element model reliably reproduces the essential mechanics of the quasi-periodic metastructures.


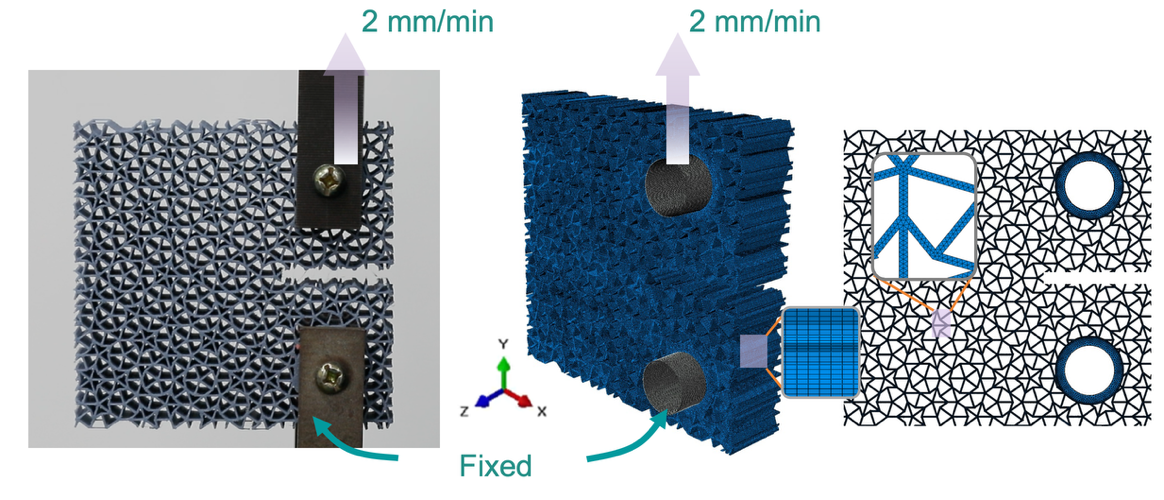


**Figure S 8.** Tensile test setup and corresponding finite-element model for the QDK specimen.


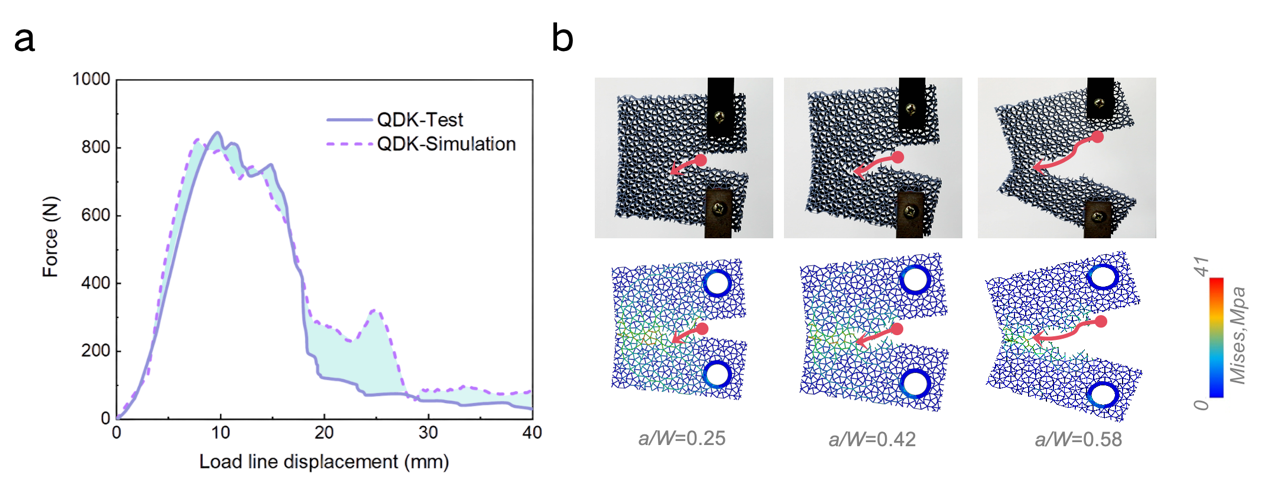


**Figure S 9.** Experimental and simulated results for the QDK specimen. **a,** Force-displacement curves. **b,** Crack-propagation sequences.

Section S6: Comparison of fracture behaviour between quasi-periodic and typical cellular structures

To further elucidate the fracture response of QDK, supplementary experiments are performed on several representative cellular structures, including square honeycomb, TPMS, and Einstein structures. All reference structures are fabricated using the same printing parameters, relative density, and control-variable strategy as those used for PH and QDK in the main text. As shown in **Figure S 10a**, QDK exhibits a higher peak load and a longer stable load-bearing stage, indicating a superior ability to sustain loading while delaying failure. By contrast, the square honeycomb and TPMS structures show a more rapid post-peak load decay, suggesting more localized crack propagation. The Einstein structure undergoes progressive deformation, but its overall load-bearing capacity remains relatively low, likely due to its 13-sided mono-tile geometry and the limited internal support it provides. Compared with the Dart-Kite motifs in QDK, the structural interactions in the Einstein architecture are weaker, resulting in less effective cooperative load bearing.

The fracture morphologies further highlight the distinct failure mechanisms of these architectures. As shown in **Figure S 10b**, the square honeycomb and TPMS structures exhibit relatively concentrated crack paths, with cracks rapidly propagating along local weak regions once initiated. In contrast, the Einstein structure shows partial crack deflection and dispersion, indicating that the introduced disorder perturbs crack propagation.


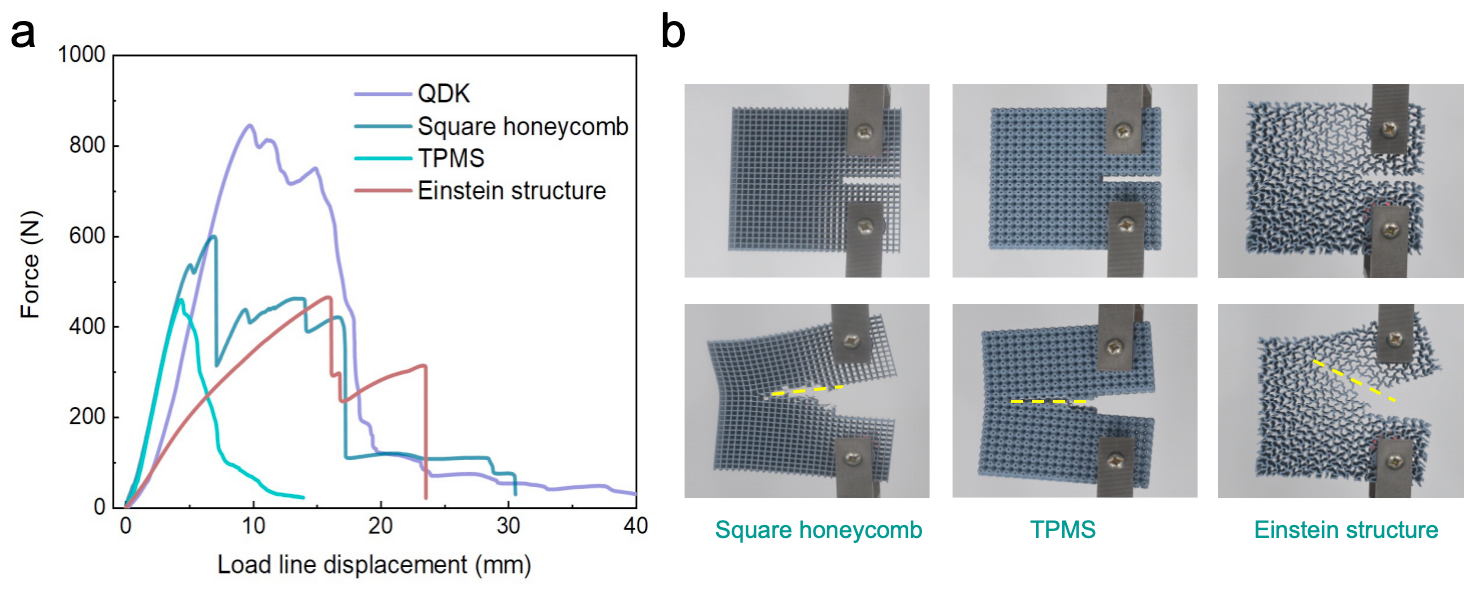


**Figure S 10.** Comparison of fracture performance among typical cellular structures. **a**, Force-displacement curves of QDK, square honeycomb, TPMS, Einstein atructures. **b**, Fractographies of QDK, square honeycomb, TPMS, Einstein atructures.

**Table S 2** summarizes the crack-path characteristics of representative cellular architectures, while **Figure 1** in the main text provides a quantitative comparison of their fracture toughness. In general, periodic structures provide clearer load-transfer paths but are more prone to localized crack propagation, whereas aperiodic structures are more effective in inducing crack deflection and path dispersion, often at the expense of load-bearing capacity. QDK is designed to bridge these two regimes by integrating geometric regularity and stable load transfer with crack-path tunability, thus mitigating the trade-off between strength and toughness while enabling predictable and tailorable crack propagation.

**Table S 2.** Comparison of fracture paths among typical cellular structures.

| Geometry | Material | Fracture mode | Type | Year |
| --- | --- | --- | --- | --- |
| 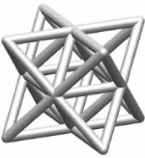 | HTL resin | 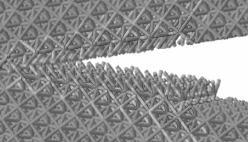 | Periodic truss | 2024^[8]^ |
| 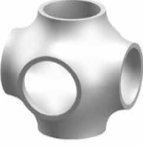 | HTL resin | 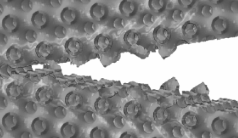 | Periodic TPMS | 2024^[8]^ |
| 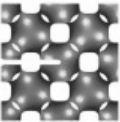 | Grey V5 photopolymer resin | 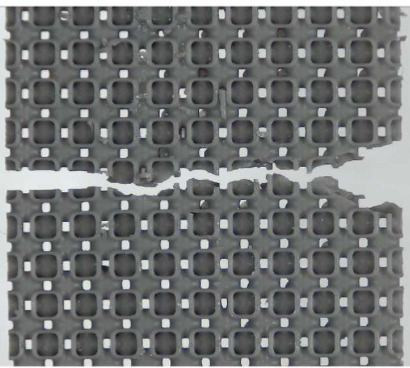 | Periodic TPMS | 2026^[15]^ |
| 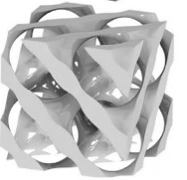 | Photopolymer (Vero) | 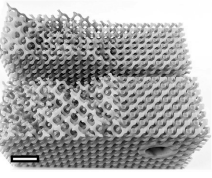 | TPMS interpenetrating-phase composite | 2023^[16]^ |
| 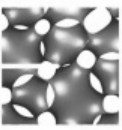 | Grey V5 photopolymer resin | 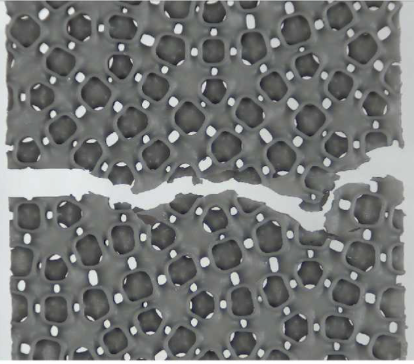 | Aperiodic TPMS | 2026^[15]^ |
| 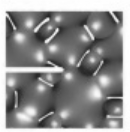 | Grey V5 photopolymer resin | 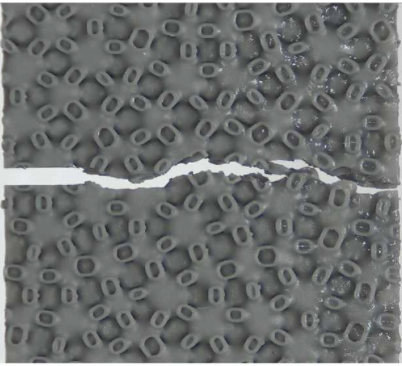 | Aperiodic TPMS | 2026^[15]^ |
| 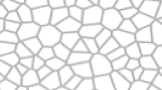 | A mixture of acrylic photopolymer and thermoplastic elastomer | 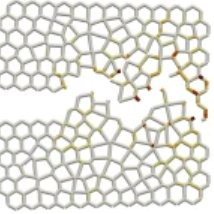 | Aperiodic Voronoi honeycomb | 2023^[17]^ |
| 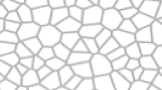 | Aluminum | 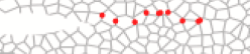 | Aperiodic Voronoi honeycomb | 2023^[18]^ |
| 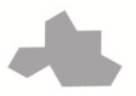 | Agilus30 Black (soft phase) + VeroClear (hard phase) | 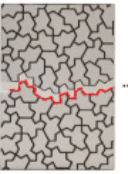 | Original Einstein tile | 2026^[19]^ |
| 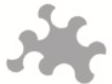 | Agilus30 Black (soft phase) + VeroClear (hard phase) | 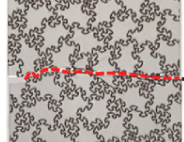 | Improved Einstein tile | 2026^[19]^ |
| *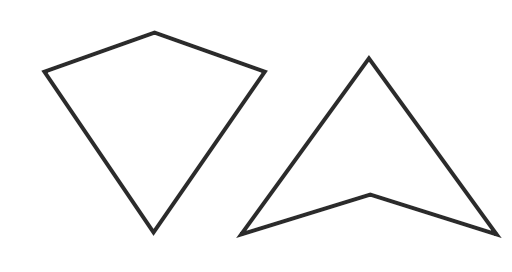* | PLA | 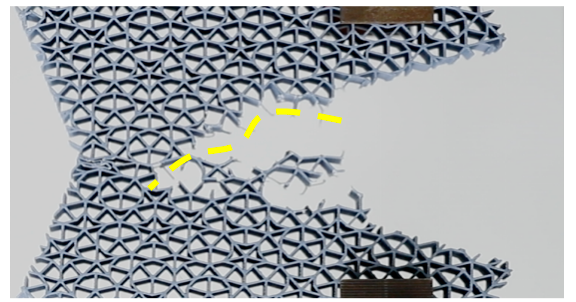 | This work | |

Section S7: Theoretical framework for linking prescribed fracture paths to J-R curves in QDK

The main text demonstrates that the fracture path of QDK can be precisely controlled through a strong-weak bond network. To correlate the prescribed fracture path with the J-R curve, a sequential fracture path model is developed based on the rupture order of the weak bonds (**Figure S 11a**). Experimental observations (**Figure 2g**) show that failure does not occur via synchronous collapse of the entire structure; rather, it initiates through successive fracture of local units and gradually evolves into a dominant crack path. The total energy dissipation can therefore be approximated as the cumulative contribution of the artificially defined weak bonds intersected by the propagating crack.


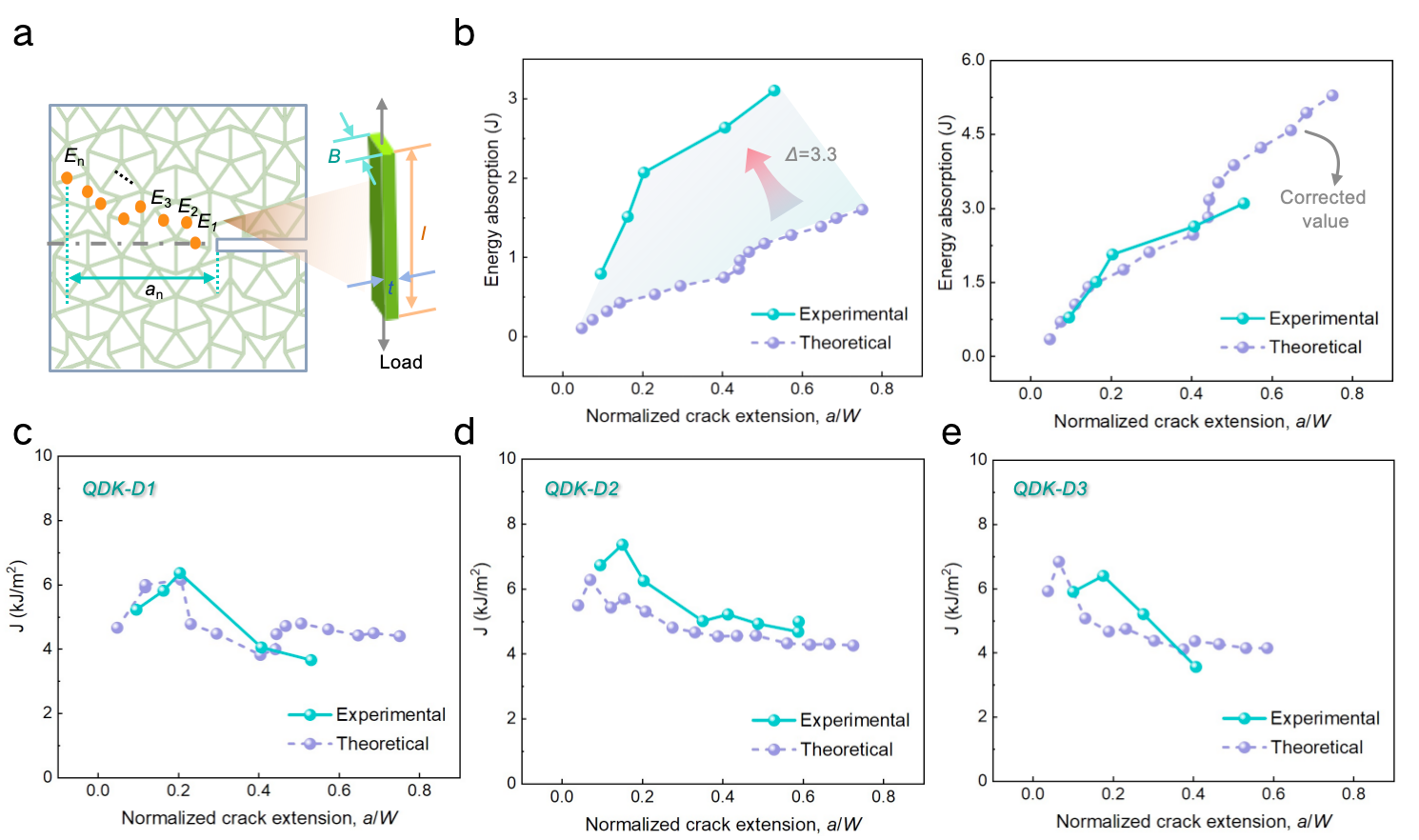


**Figure S 11.** Theoretical framework linking the prescribed fracture path to crack-growth resistance in QDK. **a**, Schematic illustration of the sequential fracture-path model. **b**, Comparison between the theoretical and experimental energy absorption as a function of normalized crack extension *a/W*. **c-e**, Comparison between the theoretical and experimental J-R curves for QDK-D1 (**c**), QDK-D2 (**d**), and QDK-D3 (**e**).

At the *n*-th fracture event, the corresponding weak bond is treated as the effective energy-dissipating element. The crack length, denoted by an, is defined as the distance from the initial crack tip to the midpoint of the *n*-th weak bond (*l* direction), and the associated dissipated energy is denoted by *E_n_*. The total fracture energy can thus be expressed as:

| $E_{f}=\sum E_{n}$ | (S11) |
| --- | --- |

This formulation recasts fracture in a relatively disordered network as the progressive failure of discrete cell-wall units along a prescribed crack path, while retaining the essential features of localized damage accumulation. Under the assumption that each load-bearing unit dissipates energy through idealized tensile fracture, the energy consumed by the *n*-th wall unit can be expressed as:

| $E_{n}=Btl\varepsilon_{b}\sigma_{s}$ | (S12) |
| --- | --- |

where *B* and *t* are the cross-sectional width and thickness of the wall, respectively, *l* is the wall length, $\sigma_{s}$ is the average stress of the base material, and $\varepsilon_{b}$ is the fracture strain (**Figure S 7d**). Accordingly, the theoretical fracture-energy evolution, $E_{f}=f(a/w)$, can be obtained, with QDK-D1 taken here as a representative case (**Figure S 11b**).

The theoretical prediction, however, shows a marked deviation from the experimental energy-*a/W* relationship. This discrepancy arises because fracture in QDK is accompanied by a pronounced plastic zone, within which substantial plastic deformation develops not only in the fractured weak-bond elements but also in the neighbouring intact walls. To account for this additional dissipation, a correction factor, *Δ*, is introduced by fitting the experimental curveas introduced by fitting the experimental curve, yielding *Δ*=3.3, so that the total dissipated energy becomes:

| $E_{total}=E_{f}\Delta$ | (S13) |
| --- | --- |

The corrected theoretical curve shows good agreement with the experimental measurements. The corresponding J-R curve calculated from the corrected energy also agrees closely with the experimental curve. Taken together, these results indicate that the present framework captures the path-dependent progressive fracture behaviour of QDK and provides a quantitative basis for relating prescribed weak-bond architectures to crack-growth resistance. This relationship further suggests that the fracture response can be estimated a priori once the weak-bond configuration is specified.

References

[1] Tang Y, Deng J, Li K, Jin M, Ng J, Li G. Quasicrystal Photonic Metasurfaces for Radiation Controlling of Second Harmonic Generation. Adv Mater. 2019;31:7.

[2] Rosa M, Karapiperis K, Radi K, Pescialli E, Kochmann D. Enhanced Deformability Through Distributed Buckling in Stiff Quasicrystalline Architected Materials. Adv Mater. 2025;37:13.

[3] Baake M, Gähler F, Mazác J, Sadun L. On the Long-Range Order of the Spectre Tilings. Discret Comput Geom. 2025:24.

[4] Qi C, Jiang F, Yang S. Advanced honeycomb designs for improving mechanical properties: A review. Composites Part B-Engineering. 2021;227.

[5] Liu J, Chen W, Hao H, Wang Z. In-plane crushing behaviors of hexagonal honeycombs with different Poisson's ratio induced by topological diversity. Thin-Walled Struct. 2021;159:13.

[6] Imediegwu C, Clarke D, Carter F, Grimm U, Jowers I, Moat R. Mechanical characterisation of novel aperiodic lattice structures q. Mater Des. 2023;229:10.

[7] A. International, ASTM E1820-24, Standard Test Method for Measurement of Fracture Toughness. 2024.

[8] Wang Y, Wu K, Zhang X, Li X, Wang Y, Gao H. Superior fracture resistance and topology-induced intrinsic toughening mechanism in 3D shell-based lattice metamaterials. Sci Adv. 2024;10:10.

[9] Zhai H, Li X, Yu S, Wang J, Chang Y, Li J et al. Review on the 3D printing technology and application of magnetic materials: Material-process-structure-application. Composites Part B-Engineering. 2025;298:40.

[10] Li Z, Wang L, Yan R, He Y, Hao H, Lin Y et al. Impact performance of curved double arrow and stacked Miura-ori panels. Eng Struct. 2025;323:13.

[11] Pei H, Yang H, Zhang N, Li T, Wang X, Zhao M et al. Moth-Wing-Inspired Multifunctional Metamaterials. Adv Mater. 2026;38:16.

[12] Clarke DJ, Imediegwu C, Jowers MI. A systematic numerical and experimental study into the mechanical properties of five honeycombs. Composites, Part B Engineering. 2023;264:110895.1–.12.

[13] Wang G, Jiao P, Bai J, Chen Z. Quasi-isotropy in non-periodic metastructures and topological solution for directional freedom. Thin-Walled Struct. 2025;214:12.

[14] A. International, ASTM D638-22, Standard Test Method for Tensile Properties of Plastics. 2022.

[15] Daynes S. Aperiodic minimal surfaces for high toughness metamaterials. Extreme Mech Lett. 2026;83:7.

[16] White B, Garland A, Boyce B. Toughening by interpenetrating lattices. Matter. 2023;6:570–82.

[17] Karapiperis K, Kochmann D. Prediction and control of fracture paths in disordered architected materials using graph neural networks. Commun Eng. 2023;2:9.

[18] Choukir S, Egmond D, Hatton B, Hibbard G, Singh C. The interplay between constituent material and architectural disorder in bioinspired honeycomb structures. Int J Eng Sci. 2023;188:16.

[19] Zhang H, Liu Y, Ma J, San Ha N, Xie YM. High-performance composites with bio-inspired interlocking aperiodic monotiles. Composites Part B: Engineering. 2026;316.
